# Photo‐Gated Corona Microfluidics

**DOI:** 10.1002/advs.76884

**Published:** 2026-07-30

**Authors:** Xiaxia Cui, Yiqing Liu, Xinyi Qiu, Manfei Liu, Biao Cheng, Yuan Zhou, Changguo Xue, Sheng Zhang, Xin Tang, Qiang Tang

**Affiliations:** ^1^ State Key Laboratory of Digital Intelligent Technology for Unmanned Coal Mining Anhui University of Science and Technology Huainan China; ^2^ Anhui University of Science and Technology Huainan Anhui China; ^3^ Southern University of Science and Technology Shenzhen Guangdong China

**Keywords:** corona discharge, multimodal manipulation, open‐surface microfluidics, phase‐change materials, photo‐gated microfluidics

## Abstract

We present a photo‐gated corona microfluidics (PGCM) platform for programmable manipulation of diverse microscale objects on open, electrode‐pattern‐free surfaces. In PGCM, near‐infrared laser irradiation locally triggers a reversible solid–liquid phase transition in the paraffin layer, switching the charge‐transport regime from diffusion‐limited to convection‐dominated and reconstructing highly confined electric‐field gradients. Quantitative charge measurements confirm that droplets acquire net positive charges via corona discharge, establishing Coulombic force as the dominant actuation mechanism. This photo‐programmable electrostatic landscape enables deterministic, non‐contact manipulation of liquid droplets, solid particles, and gas bubbles using a single movable laser beam. The platform supports comprehensive microfluidic operations—including transport, fusion, splitting, dispensing, and reaction regulation—while maintaining reliable performance on inclined, vertical, inverted, and curved surfaces. PGCM further exhibits exceptional scalability, enabling cross‐scale manipulation spanning approximately ten orders of magnitude in volume, from picoliters to milliliters, and broad compatibility with corrosive liquids and biological samples. Owing to its structurally simple architecture and unified manipulation of multiphase and cross‐scale objects, PGCM establishes a versatile framework for open microfluidics with broad applications in chemical synthesis, bioanalysis, and intelligent lab‐on‐a‐chip systems.

## Graphical abstract

Photo‐gated corona microfluidics decouples optical programming from electric‐field actuation to enable reconfigurable manipulation on open, electrode‐pattern‐free surfaces. Near‐infrared irradiation locally melts paraffin, creating a reversible charge‐relaxation pathway that reconstructs the electric field. The resulting electrostatic and electrohydrodynamic forces guide droplets, particles, and bubbles across complex geometries, spanning picoliter‐to‐milliliter volumes and supporting chemical and biological operations.

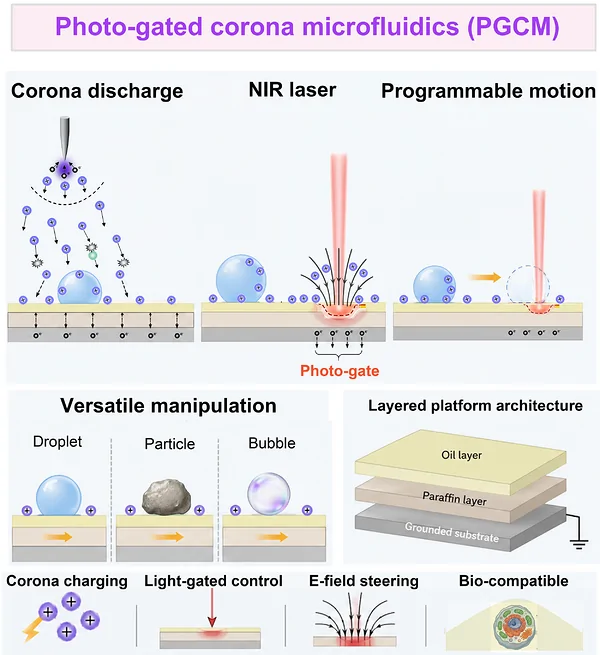

## 1 Introduction

Microfluidic technologies, by enabling precise manipulation of minute fluid volumes at the micro‐ and nanoscale, have profoundly transformed a wide range of fields, including chemical synthesis, biomedical diagnostics, drug screening, and cell analysis [1, 2, 3]. Among them, open droplet microfluidics has emerged as a powerful platform for high‐throughput and parallelized processing [4], owing to its distinctive advantages such as the elimination of channel clogging [5], facile sample access [6], and inherent compatibility with multiphase and heterogeneous reactions [7]. The core of these systems lies in the programmable, non‐contact manipulation of droplets [8], encompassing fundamental operations such as transport, positioning, merging, splitting, and dispensing [9]. At present, open droplet manipulation predominantly relies on the precise application of external physical fields [10, 11, 12, 13, 14].

A representative approach is digital microfluidics (DMF) [15], which drives droplet motion via electrowetting using pre‐patterned electrode arrays on a substrate [16]. DMF offers high control precision and ease of system integration [17]; however, its manipulation modes are inherently constrained by the predefined electrode geometries, limiting flexibility and reconfigurability [18]. In addition, the fabrication process is relatively complex and costly, posing significant challenges for large‐area implementations and rapid prototyping [19]. To circumvent these limitations, electrode‐pattern‐free strategies have been explored; for example, triboelectric charge injection enables droplet manipulation without patterned electrodes or external power supplies, achieving efficient actuation in both gas and liquid environments [20]. In parallel, optically driven droplet manipulation techniques have attracted considerable attention due to their non‐contact nature and high degree of reconfigurability [21, 22, 23, 24, 25, 26, 27]. For instance, optical tweezers enable highly precise manipulation but generate limited forces, rendering them ineffective for droplets with relatively large volumes (typically exceeding the microliter scale) [28, 29]. In contrast, strategies based on optothermal or Marangoni effects can produce stronger driving forces [30], yet they often require high‐power laser irradiation, which may induce localized overheating and thereby pose risks to thermally sensitive biological samples or solvent systems [31]. More critically, most existing approaches impose stringent requirements on substrate flatness, material properties, and the physicochemical characteristics of the manipulated liquids (e.g., electrical conductivity, surface tension, or pH), resulting in limited versatility in complex environments, multi‐material systems, and corrosive conditions [32, 33]. Consequently, the development of a new droplet actuation paradigm that features structural simplicity, highly programmable control, and broad compatibility with diverse environments and materials has become an urgent demand for advancing open microfluidics toward practical and complex applications.

Against this backdrop, we propose a new strategy termed photo‐gated corona microfluidics (PGCM), which decouples optical programming from electrical actuation. A near‐infrared laser serves as a programmable optical gate that locally triggers a reversible solid–liquid phase transition in the paraffin layer, switching the charge‐transport regime from diffusion‐limited to convection‐dominated. Coupled with corona discharge, these laser‐defined pathways dynamically redistribute space charges and reconstruct the electrostatic landscape, generating confined field gradients that predominantly drive microscale objects via Coulombic forces, assisted by dielectrophoretic and electrohydrodynamic effects. We elucidate the underlying physical mechanism of photo‐gated field reconstruction and demonstrate programmable manipulation of multiphase objects across an exceptionally broad volume range. PGCM supports integrated operations including transport, fusion, splitting, dispensing, reaction regulation, and biochemical analysis on diverse substrates. Collectively, this structurally simple, electrode‐pattern‐free framework establishes a new paradigm for programmable open microfluidics with broad implications for chemical synthesis, bioanalysis, and intelligent lab‐on‐a‐chip technologies.

## 2 Results and Discussion

As illustrated in Figure 1, we developed a PGCM platform that enables programmable manipulation of droplets and microscale objects on an open, electrode‐pattern‐free surface by optically modulating EHD boundary conditions. The device consists of a high‐voltage needle electrode positioned above a layered substrate comprising a silicone oil working layer, a paraffin phase‐change film, and a grounded stainless‐steel base. A stable positive corona discharge injects charges into the oil–droplet system, while a near‐infrared laser locally addresses the paraffin layer, dynamically defining optically controlled “photo‐gates” without patterned electrodes (see Note S1).

**FIGURE 1:**
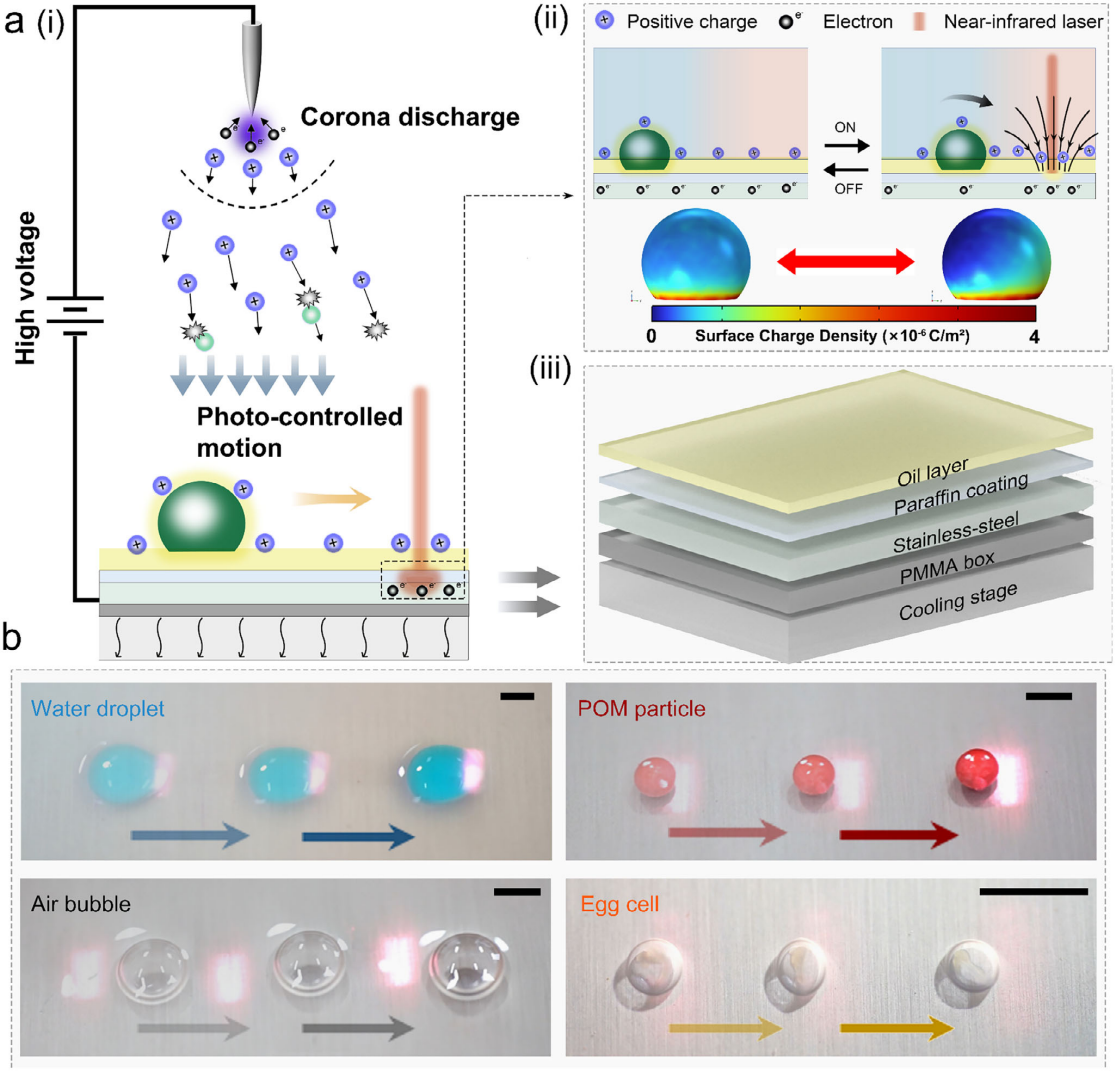
Structure, working principle, and multimodal manipulation capability of the PGCM platform. (a) Schematic of the PGCM system. (i) Overview of the experimental setup, including the high‐voltage needle electrode, the open oil‐covered surface, and the optical control path. (ii) Working principle illustrating the “photo‐gating” mechanism: a near‐infrared laser locally melts the underlying paraffin film, dynamically creating a conductive pathway that reconstructs the corona electric field and generates a trapping potential, thereby inducing redistribution of surface charges on the droplet. (iii) Exploded view of the layered platform architecture, comprising (from top to bottom) a silicone oil layer, a paraffin phase‐change film, a stainless‐steel substrate, a PMMA support chamber, and a temperature‐controlled stage. (b) Multimodal object manipulation on the PGCM platform. The platform enables programmable transport of diverse entities on the open oil surface, including a 10 µL water droplet, a ∼1 mm diameter POM solid particle, a 10 µL gas bubble, and a bio‐encapsulated sample (see Movie S1).

The operational principle is schematically illustrated in Figure 1a. In the absence of laser irradiation, the paraffin layer remains solid and highly insulating; its long dielectric relaxation time suppresses charge dissipation, maintaining a nearly uniform electric field that produces negligible net actuation. Localized near‐infrared illumination induces photothermal melting, creating a molten region with dramatically enhanced electrical conductivity relative to the surrounding solid phase. This conductivity contrast modifies the electrostatic boundary condition at the oil–substrate interface: the molten region becomes a localized low‐potential sink electrically coupled to the grounded substrate, allowing previously trapped charges to relax rapidly through the conductive pathway. Consequently, the electric potential within the illuminated zone drops sharply, establishing a steep potential gradient directed toward the laser spot. The resulting nonuniform field exerts Coulombic and dielectrophoretic forces—predominantly Coulombic—on the net‐positive‐charged droplet, deterministically pulling it toward the optically defined photo‐gate (Figure S23).

Concurrently, the phase transition switches the charge‐transport regime in the paraffin from diffusion‐limited to convection‐dominated. Coulomb body forces acting on space charges in the molten region drive electroconvection, which advects charge far more efficiently than ionic diffusion alone and reinforces the field gradient through a positive feedback loop. Meanwhile, the silicone oil layer encapsulating the droplet serves as a dielectric medium that accommodates corona‐injected space charges; under the reconstructed electric field, these charges generate electrohydrodynamic flows that supplement the direct electrostatic force via viscous momentum transfer. The oil layer also lubricates the droplet–substrate interface, reducing contact‐line pinning and motional resistance to facilitate smooth transport. Together, these coupled electrostatic and electrohydrodynamic effects enable real‐time reconfiguration of the actuation landscape via reversible phase transitions (see Note S2 and Figure S3). The photo‐gate thus functions as a dynamically addressable electrostatic trap that can be written, translated, and erased by laser scanning, while the global corona field continuously supplies the actuation energy.

The PGCM platform exhibits versatile multimodal manipulation capabilities (Figure 1b), enabling deterministic control of liquid droplets, solid particles, gas bubbles, and bio‐encapsulated samples (e.g., 3‐dpf zebrafish (*Danio rerio*) embryos) on open surfaces without surface modification. Under the unified actuation mechanism, high‐permittivity objects migrate toward the optical gate, whereas low‐permittivity gas bubbles are repelled by dielectric‐mismatch‐induced EHD flows (Figure S4), highlighting the platform's unique capability for programmable multiphase manipulation. The paraffin phase‐change layer further provides chemical robustness and long‐term stability. The platform is compatible with corrosive reagents and low‐surface‐tension liquids, sustaining repeated melting–solidification cycles without detectable performance degradation (Figures S5–S7; Movies S2 and S3). Importantly, PGCM also demonstrates short‐term compatibility under the tested PGCM conditions. Live/dead assays of HepG2 and Hep3B cells after PGCM manipulation show viabilities of ∼88%–92% across the operational voltage range, comparable to the untreated control (*p* > 0.05), accompanied by negligible pH variation and no detectable reactive species penetration (Figures S26–S28). These results confirm that the combined optical and corona‐discharge actuation preserves biological integrity during manipulation. Collectively, the unique combination of multimodal manipulation, chemical robustness, operational stability, and biocompatibility establishes PGCM as a structurally simple yet versatile platform for open microfluidics, offering broad potential for applications ranging from multiphase sample handling and chemical analysis to soft‐matter fabrication and bioanalytical systems.

To elucidate the physical origin of photo‐gated actuation, we first analyzed the laser‐induced phase transition and the resulting electric‐field reconfiguration. Infrared thermography confirms that 808 nm laser irradiation locally heats the paraffin layer above its melting point, forming a stable molten region with a characteristic radius of ∼0.8 mm (Figure 2a). Based on this physical picture, electrostatic simulations were performed to resolve the field redistribution induced by the photo‐gate. As shown in Figure 2b,c, the laser‐defined molten region reshapes the initially smooth potential landscape into a strongly nonuniform distribution, giving rise to an annular region of intensified electric field surrounding the photo‐gate, hereafter referred to as the “binding ring.” Within this annulus, charged droplets experience a net attractive force toward the gate center, whereas droplets located outside the ring are subjected to a repulsive force due to local field polarity reversal (Figures S8 and S9). This coexistence of attraction and repulsion is an intrinsic consequence of the spatial contrast in dielectric relaxation dynamics introduced by optical melting (see Note S3).

**FIGURE 2:**
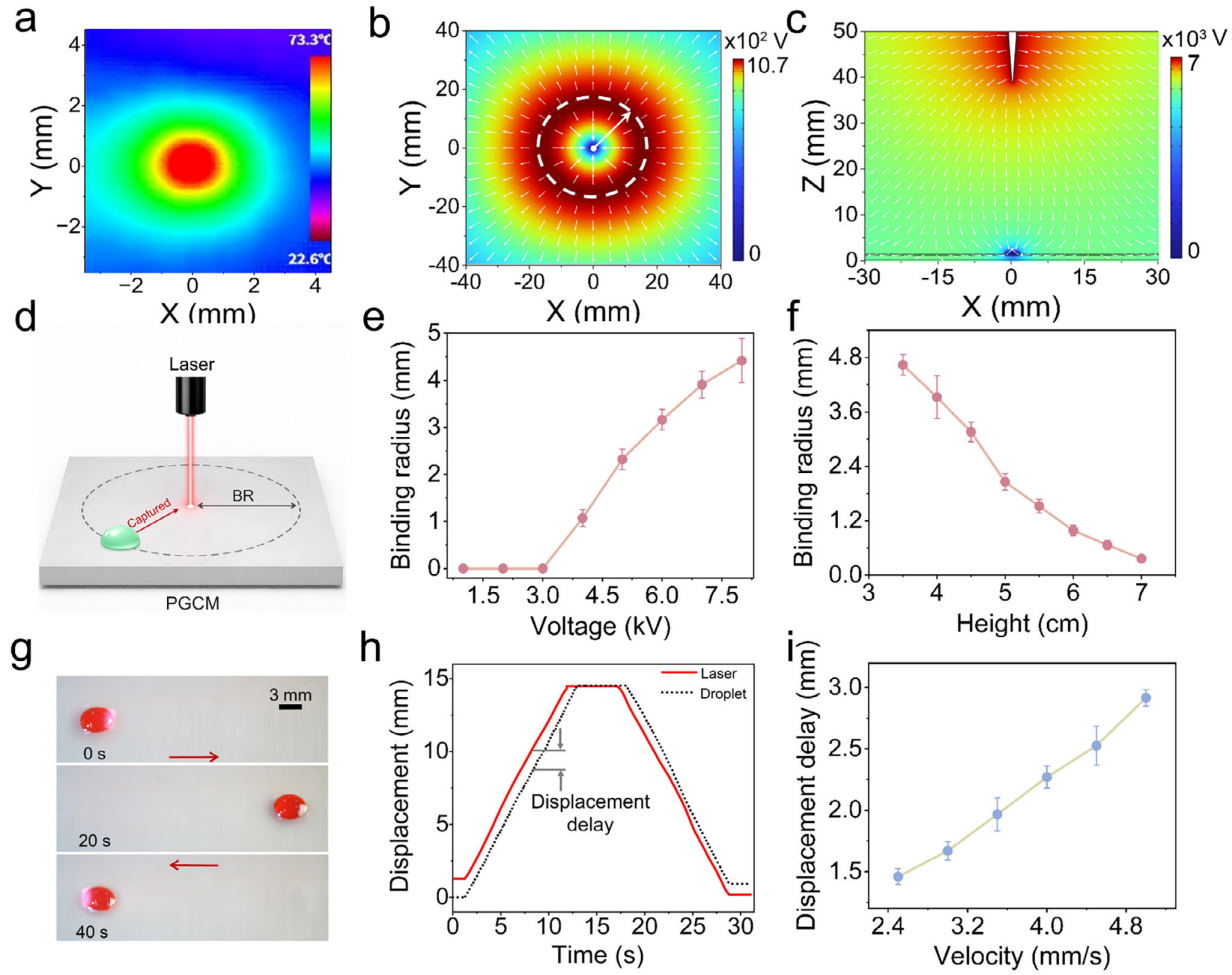
Mechanistic analysis and dynamic response characterization of PGCM. (a) Infrared thermal image of the paraffin‐coated substrate under 808 nm laser irradiation. (b) Simulated electric field line distribution on the grounded substrate surface. (c) Numerical simulation of the electrostatic potential distribution between the needle electrode and the laser‐induced molten region. (d) Schematic definition of the binding radius (BR). (e,f) Experimentally measured dependence of the binding radius BR of a 10 µL droplet on the applied voltage V and the needle‐to‐substrate separation (Height), respectively. (g) Time‐sequence images showing bidirectional transport of a 10 µL dyed droplet driven by laser scanning (see Movie S4). (h) Lateral displacement–time curves of the laser position and droplet position at an applied voltage of 5 kV; their difference is defined as the displacement delay. (i) Statistical dependence of the displacement delay on the laser scanning speed at 5 kV.

To quantify the effective range of this interaction, we define the binding radius (BR) as the maximum initial distance from which a droplet can be captured and transported toward the photo‐gate (Figure 2d). Systematic measurements reveal that BR emerges only above the corona onset voltage (∼3 kV), confirming that photothermal or Marangoni effects alone are insufficient to drive droplet motion (Figure 2e). Above this threshold, BR increases monotonically with applied voltage and decreases with increasing needle‐to‐substrate separation (Figure 2f), reflecting the tunability of the reconstructed electric field through electrical and geometric parameters. These results establish BR as a practical metric for characterizing the spatial reach and selectivity of PGCM actuation. Control experiments with reversed electrode polarity (substrate anode, needle cathode) require a substantially higher onset voltage (∼8 kV) and produce unstable splitting–fusion behavior, confirming that the standard needle‐anode configuration is physically optimized for stable actuation (Figure S25). In addition to the photo‐gate–droplet distance captured by BR; the horizontal position of the needle electrode relative to the droplet also determines actuation feasibility. We define the effective actuation radius as the maximum lateral offset from the needle axis at which a droplet can still be reliably driven, which reflects the lateral extent of the corona charge‐injection zone. This radius expands monotonically with applied voltage, ensuring robust droplet capture within the effective corona field (Figure S24).

The dynamic response of the platform was further investigated by laser scanning. Time‐sequence images and displacement–time curves (Figure 2g,h) demonstrate stable, bidirectional droplet transport in which the droplet continuously follows the moving photo‐gate. A finite displacement delay is observed due to viscous dissipation and interfacial relaxation, increasing systematically with laser scanning speed (Figure 2i). Beyond a critical speed, the droplet can no longer track the photo‐gate, indicating the existence of a maximum sustainable actuation velocity (Figure S10 and Movie S5). Notably, this velocity limit increases with applied voltage, reaching 17.6 mm s^−^^1^ at 10 kV (Figure S11). The synergistic coupling of photo‐induced phase transition, electric‐field reconstruction, and polarity‐reversal EHD enables tunable, spatially confined, and dynamically programmable manipulation of microscale objects on an open surface.

By synergistically coupling optical programming with corona‐discharge‐driven actuation, the PGCM platform performs a complete set of fundamental microfluidic operations—including transport, navigation, fusion, splitting, and dispensing—using only a single movable laser beam (Figure 3a). This unified control strategy enables fully programmable, end‐to‐end droplet manipulation on open surfaces without requiring prefabricated microchannels, patterned electrodes, or mechanical actuators, greatly simplifying system design while preserving operational flexibility. Figure 3b illustrates an integrated microfluidic workflow combining programmable transport, droplet fusion, and in situ chemical reaction. Under corona discharge, a 5 µL aqueous KSCN droplet (0.05 mol L^−^^1^) is guided by the photo‐gate toward a second 5 µL FeCl_3_ droplet (0.1 mol L^−^^1^), where rapid coalescence occurs upon contact. The immediate appearance of the characteristic blood‐red Fe(SCN)_3_ complex confirms both efficient merging and instantaneous reaction initiation. Beyond passive coalescence, continuous corona‐induced charge injection generates EHD circulation within the merged droplet, substantially enhancing internal convective mixing and accelerating reaction kinetics (Figure S12). This demonstrates that PGCM not only manipulates droplets but also actively regulates microscale transport processes and interfacial chemical reactions.

**FIGURE 3:**
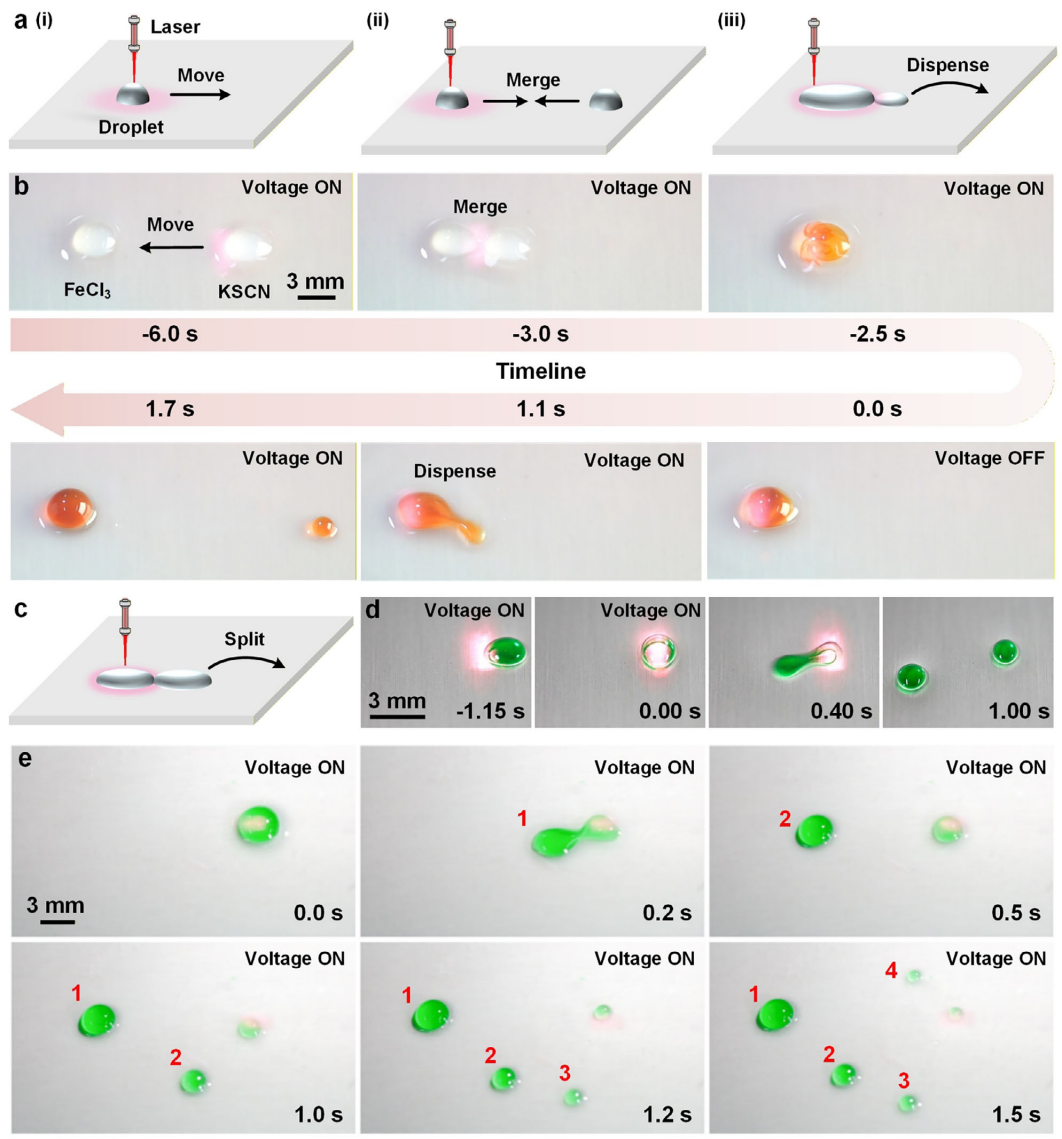
Multifunctional droplet operations enabled by PGCM. (a) Schematic illustration of the fundamental operations programmable with a single laser beam: navigation, merging, and dispensing/splitting. (b) Time‐sequence images of integrated droplet processing: A 5 µL aqueous KSCN droplet (0.05 mol L^−^^1^) is navigated by the photo‐gate toward a 5 µL FeCl_3_ droplet (0.1 mol L^−^^1^). Upon contact, the droplets merge and immediately undergo a colorimetric reaction to form red Fe(SCN)_3_. Subsequently, while maintaining laser irradiation but turning off the corona discharge, the merged droplet splits into one larger and one smaller daughter droplet (see Movie S6). (c,d) Schematic and time‐sequence images of symmetric droplet splitting achieved by positioning the laser at the droplet center (see Movie S7). (e) Time‐sequence images of recursive splitting of a residual charged droplet (20 µL) via continuous laser irradiation after the corona discharge is switched off (see Movie S8).

Beyond programmable transport and fusion, PGCM enables precise droplet dispensing and reconfiguration by decoupling optical programming from electrical actuation. When the corona discharge is switched off while laser irradiation is maintained, the laser‐induced molten paraffin region persists as a localized charge‐transport pathway. Under these conditions, the residual charges stored within the droplet interact with the reconstructed electric field, generating a pronounced lateral electric‐field gradient that induces asymmetric polarization and electrostatic stretching. Once the electrostatic stress exceeds the capillary resistance, a controlled portion of the droplet is emitted, resulting in deterministic droplet splitting (Figure S13). The splitting behavior can be tailored programmatically by adjusting the laser position relative to the parent droplet (Figure 3c,d). Irradiation near the droplet edge produces asymmetric daughter droplets owing to the off‐center electric‐field distribution, whereas irradiation at the droplet center generates a nearly axisymmetric field, leading to more symmetric division. The ejected daughter droplet consistently propagates away from the laser position, reflecting the intrinsic asymmetry of the photo‐reconstructed electrostatic landscape. A distinctive advantage of PGCM is that corona‐injected charges remain stored within the droplet for an extended period after the external voltage is removed. Consequently, multiple laser‐addressing events can be sequentially executed without reapplying the electric field, enabling recursive splitting of a single parent droplet into multiple daughter droplets with programmable volumes (Figure 3e). This capability provides a simple yet powerful strategy for on‐demand digital aliquoting and reagent dispensing.

The versatility of PGCM further extends beyond liquid manipulation to programmable soft‐material fabrication. As shown in Figure S14, sodium alginate and CaCl_2_ droplets are guided to merge under optical control, where rapid ionic crosslinking produces hydrogel bead structures in situ. This demonstration highlights the capability of PGCM to integrate programmable transport, reaction triggering, and material synthesis within a single open microfluidic platform. Collectively, these results demonstrate that PGCM provides a comprehensive, electrode‐pattern‐free microfluidic framework capable of programmable transport, fusion, splitting, dispensing, reaction regulation, and soft‐material fabrication through photo‐gated electrostatic and electrohydrodynamic control.

To evaluate the robustness and versatility of PGCM under non‐ideal and application‐relevant conditions, we systematically investigated its performance across a range of complex spatial configurations. In addition to reliable operation on inclined substrates (Figure S15), PGCM maintains stable actuation in an inverted configuration (Figure 4a). With laser irradiation from above and corona discharge applied beneath the substrate, a 5 µL water droplet can still be transported laterally beneath an ultrathin oil layer, demonstrating that droplet manipulation is independent of substrate orientation. To further challenge the platform, experiments were conducted on a vertical substrate (Figure 4b). After release, a 5 µL water droplet initially accelerates downward under gravity but is intercepted by an upward‐scanning laser within ∼90 ms. The droplet is subsequently decelerated, brought to rest, and then driven upward against gravity, achieving complete reversal of its motion. The corresponding velocity–time profile (Figure 4c) clearly captures the transition from free fall (≈ −320 mm s^−^^1^) through deceleration and zero velocity to upward acceleration. Based on the measured kinematics, the maximum laser‐induced driving force is estimated to be approximately 71 µN (Note S4), demonstrating that the reconstructed electrostatic field can readily overcome gravitational forces. Beyond single‐object manipulation, PGCM also enables programmable multi‐object operation. Multiple droplets can be independently transported within a single oil layer using a single scanning laser beam (Figure S16), while multiple laser beams enable parallel and synchronized manipulation of multiple droplets (Figure S17). This capability further supports concurrent operations such as simultaneous multi‐site detection (Figure S18), highlighting the scalability of the platform for high‐throughput and automated microfluidic applications.

**FIGURE 4:**
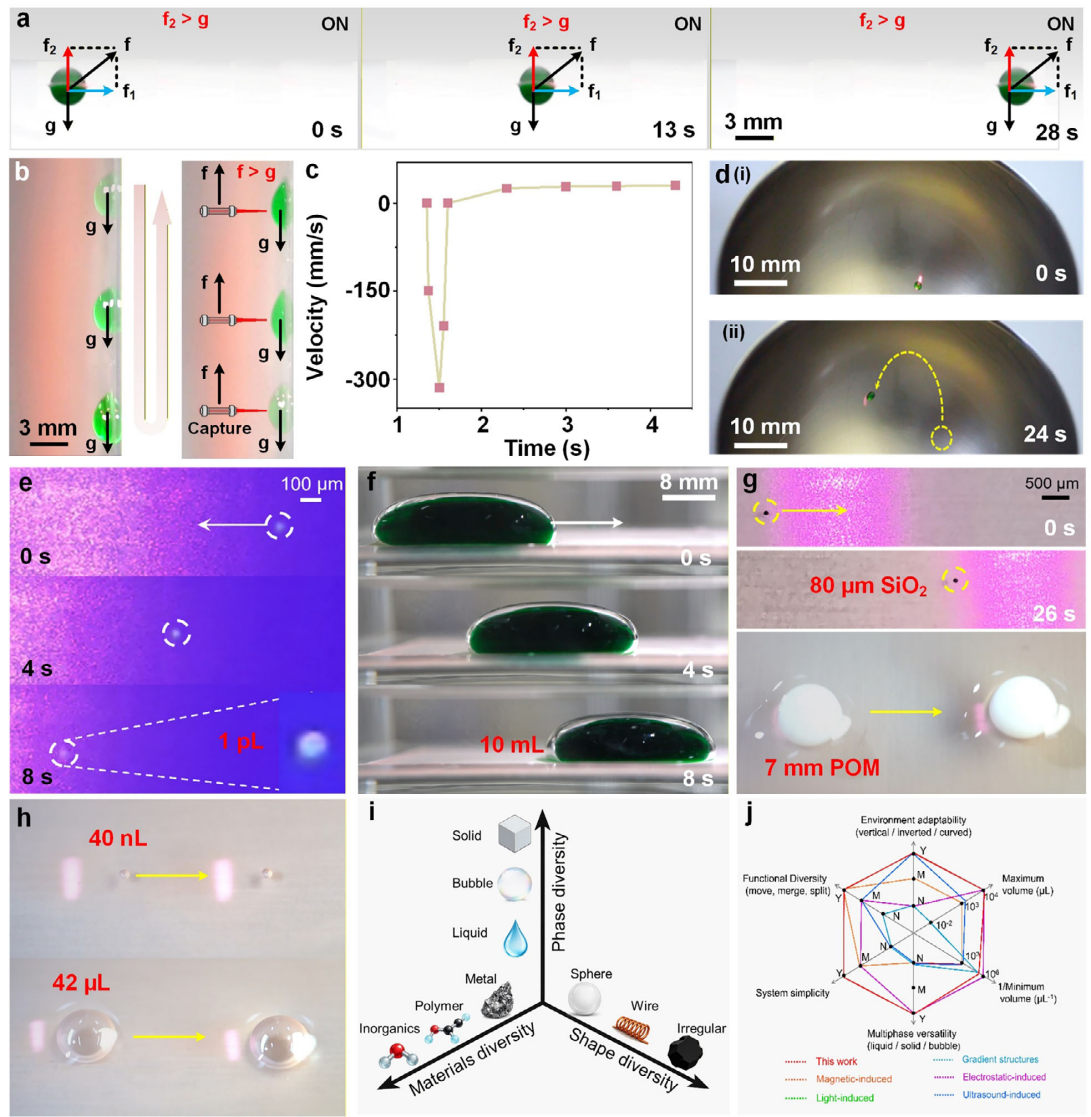
Environmental adaptability and cross‐scale manipulation capability of the PGCM platform. (a) Stable lateral transport of a 5 µL water droplet in an inverted configuration, where the needle electrode is positioned below the substrate and the laser irradiates from above (see Movie S9). (b) Capture and reverse transport of a freely falling 5 µL droplet on a vertical substrate by upward laser scanning (see Movie S10). (c) Corresponding droplet velocity–time curve for (b). (d) Guided transport of a 5 µL droplet along a predefined trajectory on a hemispherical bowl‐shaped substrate with positive Gaussian curvature (see Movie S11). (e,f) Demonstration of cross‐scale droplet manipulation, spanning volumes from the picoliter scale (1 pL) to the milliliter scale (10 mL) (see Movie S12). (g,h) Manipulation of solid particles with different sizes (diameters of 80 µm and 7 mm) and gas bubbles with different volumes (40 nL and 42 µL), respectively (see Movie S13). (i) Summary of the diversity of manipulable objects, covering different materials, physical states, and geometries. (j) Performance comparison of PGCM against five representative optically driven microfluidic techniques across five key dimensions, presented in the form of a radar chart. The ratings “Y”, “M”, and “N” correspond to “yes”, “medium”, and “no”, respectively.

The environmental adaptability of PGCM extends beyond planar substrates (Figure S19). As shown in Figure 4d, a 5 µL droplet can be guided along a predefined path on a hemispherical surface with positive Gaussian curvature (K = 494 m^−2^). Because the strategy does not rely on patterned or conformal electrodes, such controllable transport on arbitrarily curved three‐dimensional surfaces is difficult to achieve with conventional planar digital microfluidics. In terms of actuation scale, PGCM spans multiple orders of magnitude, enabling precise manipulation of picoliter droplets (1 pL; Figure 4e) and milliliter droplets (10 mL) under elevated voltage (10.5 kV; Figure 4f). The mechanism is not restricted to liquids: solid objects from ∼80 µm silica particles to 7 mm polymer bodies (Figure 4g) and gas bubbles ranging from 40 nL to 42 µL (Figure 4h) can be reliably actuated, demonstrating a unified driving principle across phases and length scales. Figure 4i summarizes the diversity of manipulable objects, including polymers, metals, and inorganic materials, spanning liquids, solids, and gas bubbles (Table S1) with varied geometries (Figure S20). Benchmarking against representative optically driven microfluidic techniques [23, 24, 27, 33] across these metrics—environment adaptability, functional diversity, system simplicity, multiphase versatility and accessible volume range (Table S2)—shows clear overall advantages of PGCM (Figure 4j). Moreover, the entire system is assembled from low‐cost, commercially available components (total material cost < 1000 CNY; Table S3), eliminating the need for expensive microfabrication facilities or complex electrode lithography. Collectively, these results position PGCM as a flexible, robust, and cost‐effective strategy for programmable manipulation on simple, open, and homogeneous surfaces without surface pretreatment or integrated electrode architectures.

Beyond its versatile capabilities for droplet manipulation, the PGCM platform also functions as a mobile sampling and pretreatment unit. As illustrated in Figure 5a, a reciprocating 5 µL aqueous droplet continuously extracts fluorescein dispersed in the surrounding oil phase, resulting in a progressive increase in fluorescence intensity that gradually approaches saturation with increasing transport distance (Figure 5b). This process enables simultaneous droplet transport and analyte enrichment without external pumps or valves. The platform further exhibits excellent multiphase compatibility: two gas bubbles (5 and 8.5 µL) can be stably captured, transported, and coalesced in a programmable manner (Figure 5c), demonstrating precise bubble merging for potential applications in multiphase microreactors, gas sensing, and interfacial engineering. Extending this capability to more complex heterogeneous environments, the PGCM platform enables selective manipulation and sorting of gas bubbles, POM particles, and water droplets under a unified actuation mechanism (Figure 5d), allowing different phases to be independently captured and delivered to predefined locations. Notably, multiple objects with distinct physical properties can be manipulated simultaneously within the same environment, underscoring the platform's versatility for integrated multiphase microfluidic operations.

**FIGURE 5:**
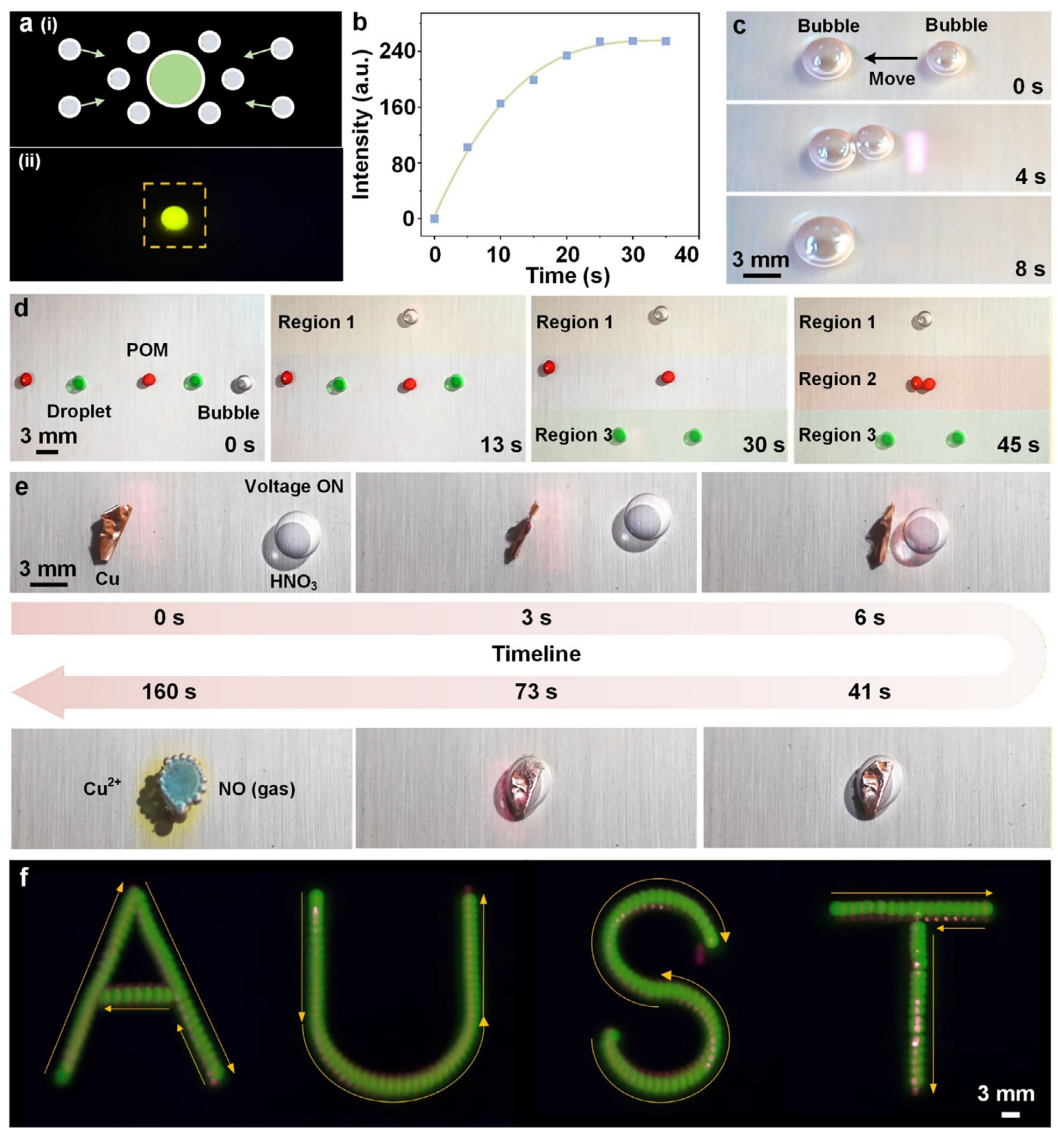
Integrated multifunctional applications enabled by PGCM. (a) Dynamic enrichment of fluorescein from the oil phase by a reciprocating 5 µL aqueous droplet, exhibiting progressively enhanced fluorescence (Movie S14). (b) Corresponding fluorescence evolution, showing time‐dependent accumulation and saturation. (c) Controlled bubble fusion between 5 and 8.5 µL bubbles (Movie S15). (d) Solid–liquid–gas three‐phase sorting, where a bubble, a POM particle, and a water droplet are selectively delivered to predefined regions (Movie S16). (e) Laser‐enhanced solid–liquid reaction: a copper foil is guided into a 5 µL nitric acid droplet, inducing rapid bubble generation and visible color change (Movie S17). (f) Programmable transport of a 2 µL fluorescent droplet to reconstruct the pattern “AUST” on an open surface without residue (Movie S18).

Beyond sampling and pretreatment, PGCM actively modulates interfacial reaction dynamics through the synergistic coupling of programmable transport, localized photothermal heating, and photo‐gated electric‐field reconstruction. As shown in Figure 5e, a ∼2.8 mm copper foil is precisely delivered into a 5 µL nitric acid droplet, initiating a heterogeneous redox reaction. Laser irradiation markedly accelerates the reaction via localized photothermal heating and photo‐gated electric‐field reconstruction at the reaction interface, producing rapid NO bubble evolution and a distinct blue Cu^2^^+^ solution. The escaping NO is subsequently oxidized to NO_2_ at the oil–gas interface (Note S5), demonstrating the platform's capability to precisely trigger, localize, and enhance microscale interfacial reactions. The high programmability of the platform is further demonstrated through precision transport tasks (Fig. S21). As illustrated in Figure 5f, a 2 µL fluorescent droplet traces predefined trajectories to reconstruct the letters “AUST” without residue, enabled by oil‐layer lubrication and photo‐gated electrostatic confinement. This precise, residue‐free patterning highlights the platform's capability for programmable routing and complex path planning. Finally, the PGCM platform integrates seamlessly with biochemical workflows. As shown in Figure S22, ninhydrin and glycine droplets are sequentially dispensed, transported, merged, and mixed to trigger a characteristic colorimetric reaction, completing an end‐to‐end workflow from reagent delivery to reaction readout without external pumps, valves, or patterned electrodes. Collectively, these demonstrations establish PGCM as an integrated microfluidic platform capable of programmable sampling, transport, reaction regulation, and biochemical analysis, providing a versatile foundation for next‐generation lab‐on‐a‐chip systems, automated microsynthesis, and intelligent chemical and biological assays.

## 3 Conclusions

In this work, we developed and experimentally validated a photo‐gated corona microfluidics (PGCM) strategy for the programmable manipulation of diverse objects on open, electrode‐pattern‐free surfaces. By decoupling optical programming from corona‐discharge‐driven actuation, PGCM provides a distinct paradigm for open microfluidics that combines the reconfigurability of optical control with the strong driving capability of electric fields. Localized near‐infrared irradiation induces reversible melting of the paraffin layer, switching it from a charge‐retentive solid state to a charge‐relaxing molten state with enhanced effective charge transport. This phase‐dependent transport contrast creates a localized low‐potential pathway coupled to the grounded substrate, dynamically redistributes interfacial charges, and reconstructs a spatially confined electric‐field landscape. The resulting field gradients exert predominantly Coulombic forces on corona‐charged droplets, with dielectrophoretic and electrohydrodynamic effects providing additional contributions to object trapping and transport. Through this mechanism, the photo‐gate functions as a dynamically writable, translatable, and erasable electrostatic trap, while the global corona field supplies the principal actuation energy.

Leveraging this mechanism, PGCM enables a broad repertoire of microfluidic operations, including transport, fusion, splitting, dispensing, enrichment, sorting, and reaction regulation, while maintaining reliable performance on inclined, vertical, inverted, and curved substrates. The platform further demonstrates exceptional scalability, spanning approximately ten orders of magnitude in manipulated volume, from picoliters to milliliters, together with unified control of liquid droplets, solid particles, and gas bubbles. Its compatibility with corrosive reagents, low‐surface‐tension liquids, physiological media, and biological samples further highlights its chemical robustness and operational versatility. Owing to its structurally simple architecture, low‐cost fabrication, real‐time reconfigurability, and broad multiphase and cross‐scale compatibility, PGCM establishes a general framework for programmable open‐surface microfluidics. Beyond conventional droplet handling, it offers a versatile basis for integrated sampling, microscale reaction control, biochemical analysis, heterogeneous object manipulation, and autonomous lab‐on‐a‐chip systems.

## 4 Experimental Section

### 4.1 Materials

Paraffin wax (melting point 58–62°C, Φ12 mm × 160 mm), silicone oil (viscosity 50 cSt), stainless‐steel sheets (100 mm × 100 mm × 0.5 mm), poly(methyl methacrylate) (PMMA) plates, anhydrous ethanol, acetone, and deionized water (18.2 MΩ·cm) were used as received. Fluorescein sodium salt, potassium thiocyanate (KSCN), iron(III) chloride (FeCl_3_), sodium alginate, calcium chloride (CaCl_2_), ninhydrin, glycine, copper foil (thickness 0.1 mm), nitric acid (HNO_3_, ∼5 mol L^−^^1^), and polyoxymethylene (POM) particles were purchased from commercial sources. Silica microspheres (diameter ∼80 µm) and other solid test objects (metals, polymers) were used for multiphase manipulation. Zebrafish embryos (*Danio rerio*, long‐finned veil‐tail mixed‐color strain, 3 days post‐fertilization) were obtained from a commercial aquaculture supplier. HepG2 and Hep3B cell lines were cultured in DMEM medium supplemented with 10% fetal bovine serum, 1% penicillin (100 U mL^−^^1^), and streptomycin (100 µg mL^−^^1^) at 37°C and 5% CO_2_.

### 4.2 Fabrication of the PGCM Platform

The layered substrate was assembled from bottom to top as follows: a temperature‐controlled stage (maintained at 20 ± 0.5°C) supported a PMMA chamber, within which a grounded stainless‐steel sheet served as both the substrate and the bottom electrode. A thin paraffin film (thickness ∼0.10–0.15 mm) was deposited on the stainless steel by melting paraffin on a hot plate at 90°C, confining it within a laser‐cut PET template, and allowing natural cooling to 20°C to form a smooth insulating layer (Figures S1 and S2). A layer of silicone oil (thickness ∼1 mm) was then spread over the paraffin film to provide an inert, lubricating boundary for droplet manipulation. A sharpened tungsten needle electrode (cone angle ∼30°, tip radius ≈ 2 µm, base diameter ≈ 350 µm) was mounted vertically above the substrate center, with its height adjustable via a micromanipulator. The needle was connected to a positive DC high‐voltage power supply (DW‐P303‐1ACD1, Dongwen High Voltage, China). The needle‐to‐substrate separation was typically set to 4.5 cm unless otherwise specified.

### 4.3 Optical and Imaging System

Localized photothermal activation was achieved using a continuous‐wave near‐infrared laser (wavelength 808 nm, power 1 W; model HXX8081000D‐AL, Tengxing Laser, China; power density 300 mW mm^−2^). The laser beam was delivered through a three‐axis translation stage to enable spatially programmable irradiation of the paraffin layer. A CMOS camera equipped with a macro lens was mounted above the platform to record top‐view videos. Side‐view imaging was performed using a second camera with a telecentric lens. An infrared thermal camera (FLIR A655sc) was used to map the temperature distribution on the substrate during laser irradiation.

### 4.4 Electrostatic Simulation

A three‐dimensional electrostatic model was established in COMSOL Multiphysics using the AC/DC Module and the Electrostatics interface (es), with the geometry constructed to reproduce the experimental configuration. The model consisted of a positively biased tungsten needle electrode positioned 45 mm above a grounded stainless‐steel substrate, a 1 mm‐thick silicone oil layer, and a 0.186 mm‐thick paraffin wax layer deposited on the substrate. A 5 µL aqueous droplet was placed at the oil–air interface, while the laser‐induced molten region in the paraffin layer was represented as a circular zone with a radius of 0.81 mm. The experimentally relevant electrical properties, including electrical conductivity and relative permittivity, were assigned to each material domain. A DC voltage of 7 kV was applied to the needle electrode, with the stainless‐steel substrate maintained at ground potential.

## Author Contributions

**Xiaxia Cui**: conceptualization, methodology, formal analysis, visualization, Writing – review and editing, Writing – original draft, funding acquisition. **Xinyi Qiu**: conceptualization, data curation, investigation, formal analysis, visualization, writing – original draft. **Yuan Zhou**: investigation, formal analysis. **Biao Cheng**: conceptualization, investigation. **Manfei Liu**: methodology, validation, visualization. **Changguo Xue**: methodology, investigation. **Qiang Tang**: methodology, validation, visualization, writing – review and editing, writing – original draft, funding acquisition, investigation, conceptualization, software, formal analysis, project administration, resources, supervision, data curation. **Xin Tang**: validation, methodology, formal analysis, conceptualization, data curation, supervision. **Sheng Zhang**: methodology, validation, writing – review and editing, writing – original draft, investigation. **Yiqing Liu**: methodology, software, investigation, validation, formal analysis, supervision, writing – original draft, visualization.

## Ethics Statement

No human participants, clinical specimens, or adult vertebrate animal procedures were involved in this study. HepG2 and Hep3B are established human cell lines. Zebrafish embryos used for qualitative transport demonstrations were commercially sourced and examined at 3 days post‐fertilization. All biological materials were handled in accordance with applicable institutional guidelines.

## Conflicts of Interest

The authors declare no conflicts of interest.

## Supporting information




**Supporting File 1**: advs76884‐sup‐0001‐SuppMat.docx.

**Supporting File 2**: advs76884‐sup‐0002‐MovieS1‐S18.zip.

## Data Availability

The data that support the findings of this study are available from the corresponding author upon reasonable request.
