# Supplementary material for: Photo‐Gated Corona Microfluidics
Source: Adv Sci (Weinh). 2026 Jul 30:e76884. Online ahead of print. doi: 10.1002/advs.76884 (PMC13423506; doi:10.1002/advs.76884)
Supplement: Supplementary file 1 — Supporting File 1: advs76884‐sup‐0001‐SuppMat.docx. [file ADVS-9999-e76884-s002.docx]

Supporting Information

**Photo-Gated Corona Microfluidics**

Xiaxia Cui^1,2&^, Yiqing Liu^2&^, Xinyi Qiu^2&^, Manfei Liu^2^, Biao Cheng^2^, Yuan Zhou^2^, Changguo Xue^2^, Sheng Zhang^2*^, Xin Tang^3*^, Qiang Tang^1,2*^

^1^ State Key Laboratory of Digital Intelligent Technology for Unmanned Coal Mining, Anhui University of Science and Technology, Huainan 232001, China

^2^ Anhui University of Science and Technology, Huainan, Anhui 232000, China

^3^ Southern University of Science and Technology, Shenzhen 518055, Guangdong, China

^&^X.C., Y.L. and X.Q. contributed equally to this work.

^*^To whom correspondence should be addressed.

**This PDF file includes:**

Supplementary Note S1 to S5

Figures S1 to S29

Table S1 to S3

Legends for Movies S1 to S18

**Other supplementary materials for this manuscript include the following:**

Movies S1 to S18

**Supplementary Note S1. Device Configuration and Experimental Parameters**

**S1.1 High-Voltage Electrode Geometry and Electrical Configuration**

The corona discharge is generated using a tungsten needle electrode positioned above the oil-covered platform. The needle has a cone angle of approximately 30°, a tip radius of ≈2 μm, and a base diameter of ≈350 μm, providing a stable and spatially confined electric field for charge injection. A stainless-steel plate serves as the grounded bottom electrode. The high voltage is supplied by a DC power source (DW-P303-1ACD1, Dongwen High Voltage, China), operating in the kilovolt range under microampere-level current conditions.

These parameters are provided for reproducibility and do not impose strict constraints on platform operation; similar geometries capable of sustaining stable corona discharge are expected to yield comparable behavior.

**S1.2 Substrate Materials and Layered Architecture**

The grounded substrate is coated with a thin paraffin phase-change layer (~0.10–0.15 mm thickness, Figs. S1 and S2), which functions as an optically switchable charge-transport boundary. Stainless steel is employed as the base electrode due to its high thermal conductivity and mechanical robustness, enabling rapid heat dissipation during repeated phase transitions. A silicone oil layer (~1 mm thickness, viscosity 50 cSt) covers the paraffin surface and serves as the working dielectric medium. The silicone oil provides high electrical resistivity, chemical stability, and favorable interfacial properties for encapsulating diverse micro-objects. These characteristics support stable electrohydrodynamic operation without requiring patterned electrodes or specialized surface treatments. In addition to encapsulating and protecting droplets, the oil layer enhances the effective electrostatic coupling to the droplet and reduces motional resistance through lubrication.

**S1.3 Temperature Control and Phase-Change Regulation**

The entire device is mounted on a temperature-controlled stage maintained at approximately 20 °C. This baseline temperature ensures rapid and reversible solid–liquid transitions of the paraffin layer following localized laser heating, while providing active heat dissipation that suppresses droplet evaporation and minimizes thermal damage to biological samples (Fig.S29).

**S1.4 Optical Excitation Parameters**

Localized photothermal activation is achieved using a continuous-wave near-infrared laser (wavelength 808 nm, power 1 W; model HXX8081000D-AL, Tengxing Laser, China; power density 300 mW mm⁻²). The laser beam is delivered through a three-axis translation stage to enable spatially programmable irradiation of the paraffin layer. Detailed information regarding laser model and alignment is provided here solely for experimental reproducibility; the photo-gating mechanism is not dependent on a specific commercial source.

**S1.5 Electrical Power Characteristics**

During operation, the PGCM platform functions under a low-power corona discharge regime. The measured current is on the order of microamperes (Keithley 6517B), corresponding to milliwatt-level electrical power consumption. This low energy requirement reflects the fact that optical excitation modulates charge transport pathways rather than directly supplying mechanical actuation energy.

**Supplementary Note S2: Physical Origin of Photo-Gated Charge Transport Switching in the PGCM Platform**

**S2.1 Phase-Dependent Charge-Transport Framework**

The near-infrared photo-gate in the PGCM platform does not induce an intrinsic electronic insulator-to-conductor transition in paraffin. Instead, laser-induced melting changes the mobility of charge carriers and enables fluid motion within the phase-change layer, thereby altering the dominant mechanisms and characteristic timescale of charge transport.

The evolution of the local space-charge density, $\rho_{e}$, can be described by the charge-conservation equation:

$$\begin{aligned} \begin{matrix} \frac{\partial\rho_{e}}{\partial t}+\nabla\cdot\mathbf{J}=0 \end{matrix}\#\left( S2.1 \right) \end{aligned}$$

where the total charge flux $\mathbf{J}$ can be expressed phenomenologically as

$$\begin{matrix} \mathbf{J}=\sigma\mathbf{E}-D\nabla\rho_{e}+\rho_{e}\mathbf{u}. \end{matrix}$$

Here, $\sigma\mathbf{E}$ represents field-driven conduction, $D\nabla\rho_{e}$ represents charge diffusion, and $\rho_{e}\mathbf{u}$ represents charge advection by local fluid motion. The relative contributions of these terms depend strongly on whether the paraffin is in the solid or molten state.

Accordingly, the photo-gating process switches the system between two distinct charge-transport states:

- **OFF state—solid paraffin:** charge relaxation is strongly suppressed because of the extremely low electrical conductivity and the absence of bulk fluid motion.
- **ON state—molten paraffin:** enhanced ionic mobility and field-induced fluid motion substantially accelerate local charge transport and relaxation.

This phase-dependent contrast dynamically changes the electrical boundary condition at the oil–paraffin–substrate interface and provides the physical basis for photo-programmable electric-field reconstruction.

**S2.2 OFF State: Charge-Retentive Solid Paraffin**

Without laser irradiation, the paraffin layer remains solid and highly insulating. Its very low conductivity results in a long dielectric relaxation time,

$$\begin{aligned} \begin{matrix} \tau_{d}=\frac{\varepsilon}{\sigma} \end{matrix}\#\left( S2.2 \right) \end{aligned}$$

where $\varepsilon$ and $\sigma$ are the permittivity and electrical conductivity of the paraffin, respectively. When $\tau_{d}$ is much longer than the experimental timescale, injected charges cannot rapidly relax through the paraffin layer to the grounded stainless-steel substrate.

In addition, the solid matrix prevents bulk fluid motion, such that $\mathbf{u}\approx0,$and the advective term in Equation (S2.2) vanishes. Charge transport is therefore restricted to the weak conduction and diffusion processes permitted by the solid dielectric. Consequently, the interfacial charge distribution changes only slowly, and no sharply localized lateral potential gradient is established in the absence of optical activation.

The solid paraffin layer thus functions as a **charge-retentive boundary**, preserving the pre-existing electrostatic state rather than providing a localized pathway for rapid charge dissipation.

**S2.3 ON State: Convection-Assisted Charge Relaxation in Molten Paraffin**

Under localized 808 nm laser irradiation, the paraffin temperature rises above its melting point and a spatially confined molten region is formed. Melting increases molecular and ionic mobility and markedly reduces the mechanical resistance to fluid motion. As a result, both field-driven conduction and convection-assisted charge transport become possible within the illuminated region.

Space charges in the molten paraffin experience a Coulomb body-force density given by

$$\begin{aligned} \begin{matrix} \mathbf{f}_{e}=\rho_{e}\mathbf{E} \end{matrix}\#\left( S2.3 \right) \end{aligned}$$

This electrical body force can generate local electrohydrodynamic motion, or electroconvection, within the fluidized region. The resulting velocity field introduces an advective charge flux, $\rho_{e}\mathbf{u}$, in addition to conventional conductive and diffusive transport. Charge can therefore be transported toward and relaxed through the grounded substrate more rapidly than in the surrounding solid paraffin.

For convenience, the combined effects of conduction and flow-assisted charge transport can be represented by an effective conductivity, $\sigma_{\mathrm{eff}}$, and a corresponding effective relaxation time:

$$\begin{aligned} \begin{matrix} \tau_{\mathrm{eff}}=\frac{\varepsilon}{\sigma_{\mathrm{eff}}} \end{matrix}\#\left( S2.4 \right) \end{aligned}$$

Here, $\sigma_{\mathrm{eff}}$ should be understood as a phenomenological parameter describing the overall charge-transport capability of the molten region, rather than the intrinsic electronic conductivity of paraffin. Because charge transport is enhanced after melting,

$\sigma_{\mathrm{eff},\mathrm{molten}}>\sigma_{\mathrm{eff},\mathrm{solid}},$and therefore$\tau_{\mathrm{eff},\mathrm{molten}}<\tau_{\mathrm{eff},\mathrm{solid}}.$The illuminated molten region consequently behaves as a rapidly relaxing, locally low-potential pathway that is electrically coupled to the grounded substrate. In contrast, the surrounding solid paraffin retains charges over a much longer timescale. This spatial difference in charge-relaxation dynamics produces a steep lateral potential variation at the boundary between the molten and solid regions.

**S2.4 Experimental Evidence for Reversible Transport Switching**

The phase-dependent change in charge transport is directly reflected in the measured electrical current of the PGCM system. As shown in Fig. S3, at an applied voltage of 6 kV, the current remains at approximately 0.25 μA before laser irradiation. After the laser is turned on, the current gradually increases to approximately 0.32 μA as the paraffin melts. When the laser is subsequently switched off, the current decreases and stabilizes at approximately 0.24 μA as the paraffin resolidifies.

The reversible current increase provides experimental evidence that the molten region supports more efficient charge transfer than the solid paraffin. The gradual temporal response is also consistent with the finite times required for local melting, development of convection-assisted transport, and subsequent resolidification. Although the current measurement does not independently separate conductive and advective contributions, it confirms that laser irradiation reversibly increases the effective charge-transport capability of the phase-change layer. The observed response therefore supports the proposed switching between a charge-retentive solid state and a charge-relaxing molten state.

**S2.5 Connection to Electric-Field Reconstruction and Droplet Actuation**

The photo-gating effect originates from the spatial coexistence of these two transport states. Inside the laser-irradiated region, accelerated charge relaxation lowers the local electric potential, whereas the surrounding solid paraffin retains charge and sustains a comparatively higher potential. The resulting lateral potential gradient reconstructs the electric field near the oil–substrate interface and generates an annular region of enhanced field intensity around the photo-gate, as further discussed in Supplementary Note S3.

This reconstructed field exerts a Coulombic force on corona-charged droplets,$\begin{matrix} \mathbf{F}_{C}=q\mathbf{E}, \end{matrix}$ and a secondary dielectrophoretic force associated with the field gradient. For the net positively charged aqueous droplets employed in the PGCM system, Coulombic attraction is the dominant contribution that determines the direction of motion. Electrohydrodynamic flow in the surrounding silicone oil further assists transport by transferring viscous momentum to the droplet and reducing the effective resistance to motion.

Therefore, the laser functions primarily as a spatially programmable **gate for charge relaxation and electric-field reconstruction**, whereas the corona-generated electric field supplies the principal actuation energy. Reversible melting and resolidification allow the electrostatic landscape to be dynamically written, translated, and erased without patterned electrodes.

**Supplementary Note S3: An Idealized Physical Model for the Formation of the Binding Ring**

The formation of the annular electrostatic trap (“binding ring”) observed in PGCM experiments can be captured by a semi‑analytical model that treats the laser‑irradiated region as a localized perturbation in the effective charge‑transport properties of the paraffin layer.

**S3.1 Model Setup: Spatially Modulated Effective Conductivity**
We approximate the system in a quasi‑static limit, where the electric potential ϕ*ϕ* satisfies the charge‑conservation equation:

$$\begin{aligned} \begin{matrix} \nabla\cdot[\sigma_{\text{eff}}(\mathbf{r})\nabla\phi(\mathbf{r})]=0, \end{matrix}\#\left( S3.1 \right) \end{aligned}$$

where *σ*_eff_​(**r**) represents the effective conductivity of the paraffin layer, which switches sharply between two distinct regimes upon laser illumination:

$$\begin{aligned} \begin{matrix} \sigma_{\text{eff}}(r)\approx\{\begin{matrix} \sigma_{m}, & r\leq r_{0}\text{(molt}\text{en, high‑transport state)}, \\ \sigma_{s}, & r>r_{0}\text{(solid, insulating state)}. \end{matrix} \end{matrix}\#\left( S3.2 \right) \end{aligned}$$

Here *r*_0​_ is the radius of the laser spot, $\sigma_{m}\gg\sigma_{s}$, and the transition is treated as a step‑like discontinuity for clarity. The large contrast $\sigma_{m}/\sigma_{s}$ (several orders of magnitude) reflects the dramatic difference in charge‑relaxation times between the molten ($\tau_{m}=\varepsilon/{\sigma_{m}}$​) and solid ($\tau_{s}=\varepsilon/{\sigma_{s}}$​) states.

**S3.2 Field Redistribution and the Emergence of a Radial Maximum**
Solving Eq. (S3.1) with the boundary condition (S3.2) shows that the electric‑field magnitude ∣E∣=∣−∇ϕ∣ does **not** peak at the center of the laser spot. Instead, it reaches a maximum in a narrow annular region near the conductivity discontinuity at $r\approx r_{0}$. This occurs because the molten core ($r<r_{0}$​) acts as a **dynamic charge‑relaxation sink**, rapidly draining space charge to the grounded substrate and thus suppressing the internal field. In contrast, charge accumulates in the solid region ($r>r_{0}$) due to its long relaxation time. The resulting mismatch in charge‑relaxation rates creates a steep lateral gradient in the electrostatic potential, which manifests as a ring‑shaped region of intensified electric field—the **binding ring**.

**S3.3 Physical Interpretation and Role of Electroconvection**
Physically, the binding ring arises from a **light‑defined spatial contrast in dielectric relaxation dynamics**:

- **Inside the laser spot:** $\tau_{m}\ll t_{\text{flow}}$; charge relaxes almost instantaneously, making the region equipotential.
- **Outside the laser spot:** $\tau_{s}\gg t_{\text{flow}}$​; charge is effectively frozen, sustaining a higher potential.

The sharp transition between these two regimes forces the equipotential lines to compress radially at $r\approx r_{0}$​, producing a localized electric‑field maximum. In practice, **electroconvection** within the molten zone continuously supplies fresh space charge to the boundary, reinforcing the field gradient and stabilizing the ring against charge depletion.

**S3.4 Implications for PGCM Operation**

This model clarifies that the binding ring is not predefined by electrode patterns or material heterogeneity. It is a **self-organized electrohydrodynamic structure** that emerges dynamically from the optical modulation of charge-transport boundaries. The molten core acts as a **localized low-potential sink**, and the steep potential gradient at $r\approx r_{0}$​ generates the Coulombic attractive force that pulls net-charged droplets toward the laser-defined gate.

The ring's strength and position can be tuned in real time by adjusting the laser spot (size and location) and the corona field (voltage), providing the spatial programmability that underlies all PGCM droplet manipulation functions. The semi-analytical approach therefore bridges the microscopic charge-relaxation picture (Note S2) and the macroscopic droplet motion observations, offering a unified explanation for the platform's reconfigurable trapping capability.

**Supplementary Note S4: Estimation of the maximum laser driving force**

**Sign convention.** The upward direction is defined as positive. The droplet velocity v(t) is obtained from video tracking, and the acceleration is computed as

$$\begin{aligned} a\left( t \right)=\frac{dv\left( t \right)}{dt}\#\left( S4.1 \right) \end{aligned}$$

The peak upward acceleration during the capture/turning stage is defined as

$$\begin{aligned} a_{max}={max}_{t}\left( \frac{dv(t)}{dt} \right)\#\left( S4.2 \right) \end{aligned}$$

**Force balance.** Along the vertical direction, the 1D force balance can be written as

$$\begin{aligned} F_{\text{laser}}(t)-mg-F_{\text{d}}(t)=m\text{ }a(t)\#\left( S4.3 \right) \end{aligned}$$

where $m$ is the droplet mass, $g$ is the gravitational acceleration, and $F_{\text{d}}(t)$ represents dissipative resistance (e.g., viscous shear/contact-line dissipation) opposing the instantaneous motion.

For a conservative “up to” estimate, we neglect the dissipative term and obtain

$$\begin{aligned} F_{\text{laser}}\left( t \right)\approx m \left[ g+a\left( t \right) \right]\#\left( S4.4 \right) \end{aligned}$$

Therefore, the maximum driving force is estimated as

$$\begin{aligned} F_{max}={max}_{t}F_{\text{laser}}(t)\approx m\text{ }[g+a_{max}]\#\left( S4.5 \right) \end{aligned}$$

**Mass definition.** The droplet mass is determined by

$$\begin{aligned} m=\rho V\#\left( S4.6 \right) \end{aligned}$$

where $V$=5 μL is the droplet volume and $\rho$ is the droplet density.

Using the experimentally extracted $a_{max}$ from the $v(t)$ curve and the above relation, the maximum laser driving force is calculated to be **up to ∼71 μN.**

**Supplementary Note S5:**

In this experiment, an irregular copper foil (≈ 2.8 mm in length) was first transported by the PGCM system and brought into contact with a 5 µL droplet of dilute nitric acid (∼ 5 mol·L⁻¹, below the 6 mol·L⁻¹ threshold for concentrated acid).

For dilute nitric acid, the classic redox reaction with copper proceeds moderately at room temperature, primarily producing colorless nitric oxide (NO) gas. Correspondingly, the primary reaction equation is:

$$\begin{aligned} 3\text{Cu}+8\text{H}^{+}+2\text{NO}_{3}^{-}\to3\text{Cu}^{2+}+2\text{NO}\uparrow+4\text{H}_{2}\text{O}\#\left( S5.1 \right) \end{aligned}$$

Consistent with this mechanism, the initial reaction in the absence of laser irradiation was slow, with minimal bubble generation. However, upon applying near-infrared laser illumination to the reaction interface, the synergistic effects of **localized photothermal heating** and **photo-gated electric-field reconstruction** dramatically accelerated the reaction kinetics. This enhancement was evidenced by the rapid evolution of numerous gas bubbles from the copper surface and a distinct color change of the droplet to blue, confirming the accelerated formation of Cu²⁺ ions.

The generated NO gas subsequently underwent oxidation at the oil-gas interface by ambient oxygen, yielding nitrogen dioxide (NO₂), which appeared as yellow-brown fumes:

$$\begin{aligned} 2\text{NO}+\text{O}_{2}\to2\text{NO}_{2}\#\left( S5.2 \right) \end{aligned}$$

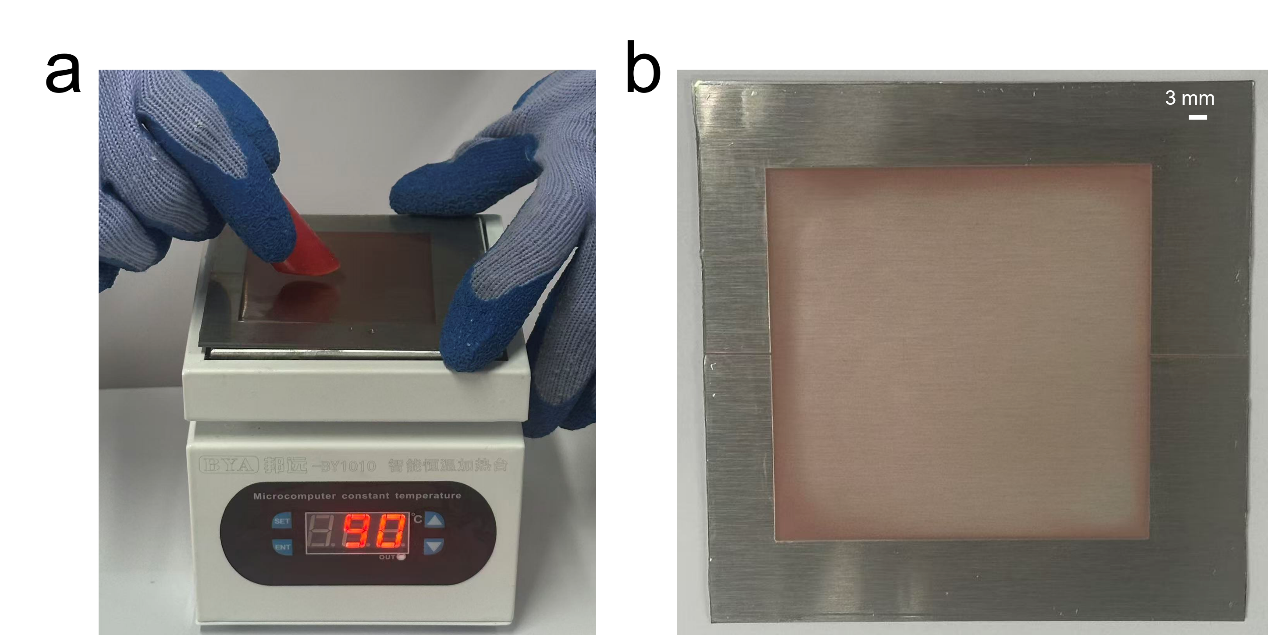


**Fig. S1. Fabrication process of the paraffin phase-change layer.** (a) Schematic illustration of the wax-coating procedure. A square confinement region is first defined by covering a stainless-steel plate with a 0.5-mm-thick PET film and patterning it using laser cutting, ensuring that the molten paraffin is restricted within the designated area. The substrate is then placed on a temperature-controlled heating stage maintained at 90 °C, and paraffin is gently applied and uniformly spread within the confined region using a candle. After coating, the temperature is lowered to 20 °C and the sample is allowed to cool naturally. (b) Photograph of the substrate after completion of the paraffin coating.


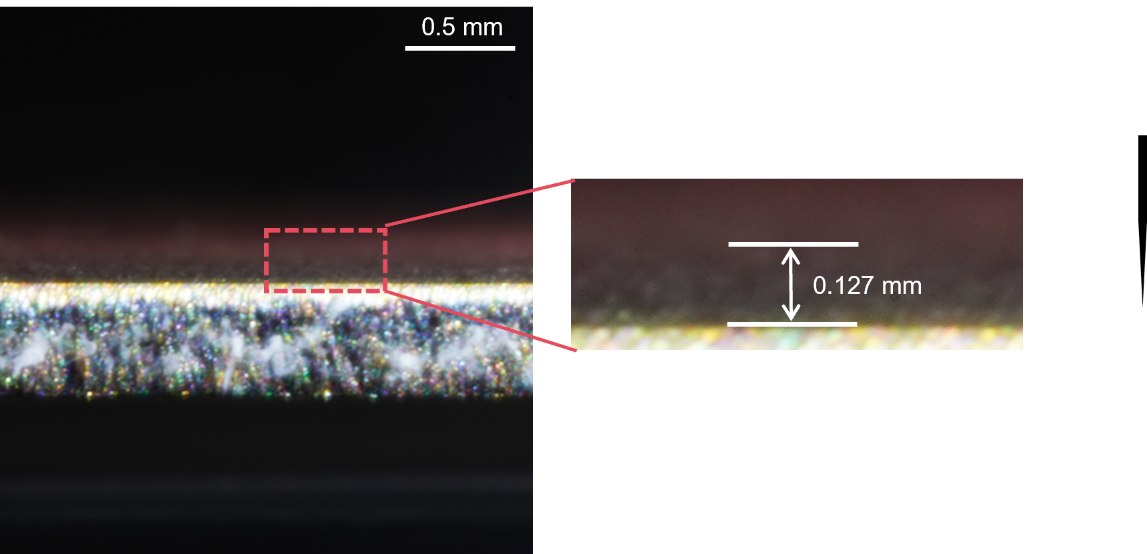


**Fig. S2.** Microscopic cross-sectional image of the paraffin layer coated on the stainless-steel substrate, showing a uniform wax thickness of approximately 127 µm.


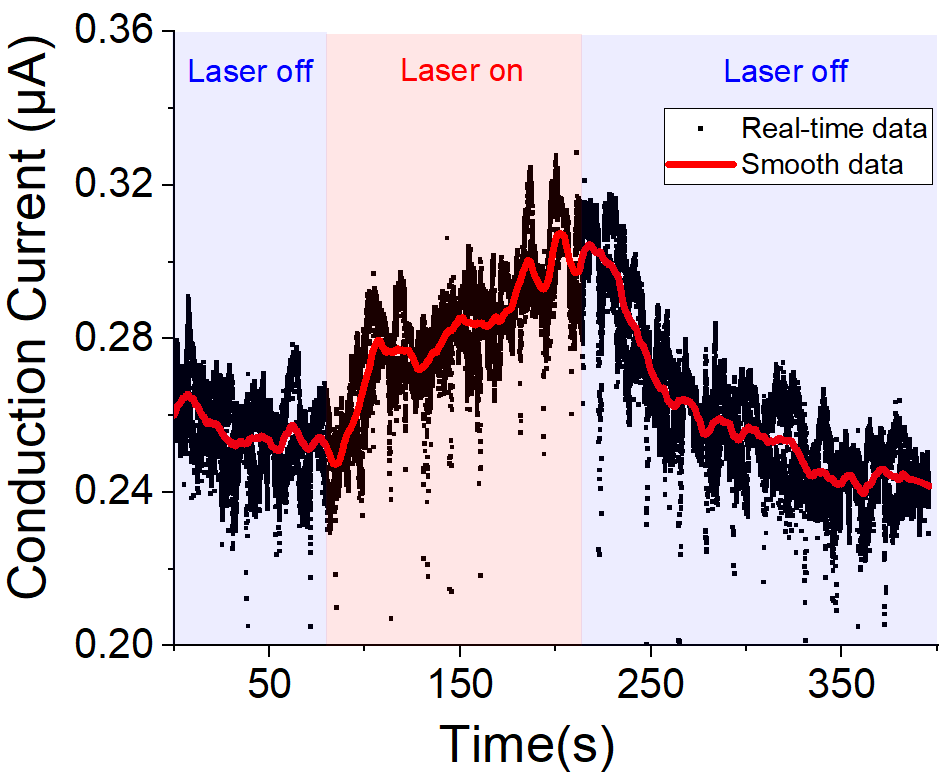


**Fig. S3. Current response of the PGCM system measured using a Keithley 6414 electrometer under an applied voltage of 6 kV.** With the laser OFF, the current remains stable at ≈0.25 μA, consistent with a long dielectric relaxation time ($\tau_{s}$​) in the solid paraffin state, where charge transport is strongly suppressed. Upon laser irradiation, progressive melting of the paraffin layer activates electrohydrodynamic charge transport, leading to a gradual increase in current up to ≈0.32 μA. After the laser is turned OFF, resolidification restores a diffusion-limited regime, and the current decreases and stabilizes at ≈0.24 μA. The reversible current modulation reflects laser-induced switching between charge-restrictive and convection-enhanced transport states, in agreement with the timescale analysis described in Note S1.


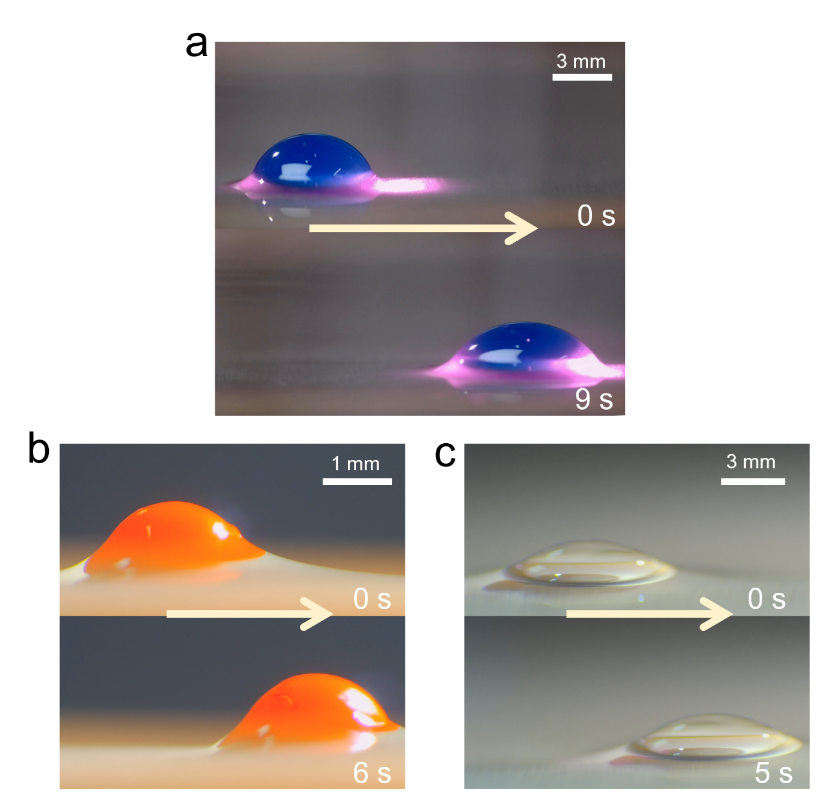


**Fig. S4. Side-view visualization of droplet, solid particle, and bubble actuation on the PGCM platform.** (a) Transport of a 10 µL liquid droplet under a thin lubricating oil layer (silicone oil viscosity: 50 cSt) at an applied needle voltage of 6 kV. (b) Directed motion of a solid POM particle with a diameter of approximately 1 mm. (c) Actuation of a gas bubble with a volume of approximately 10 µL.


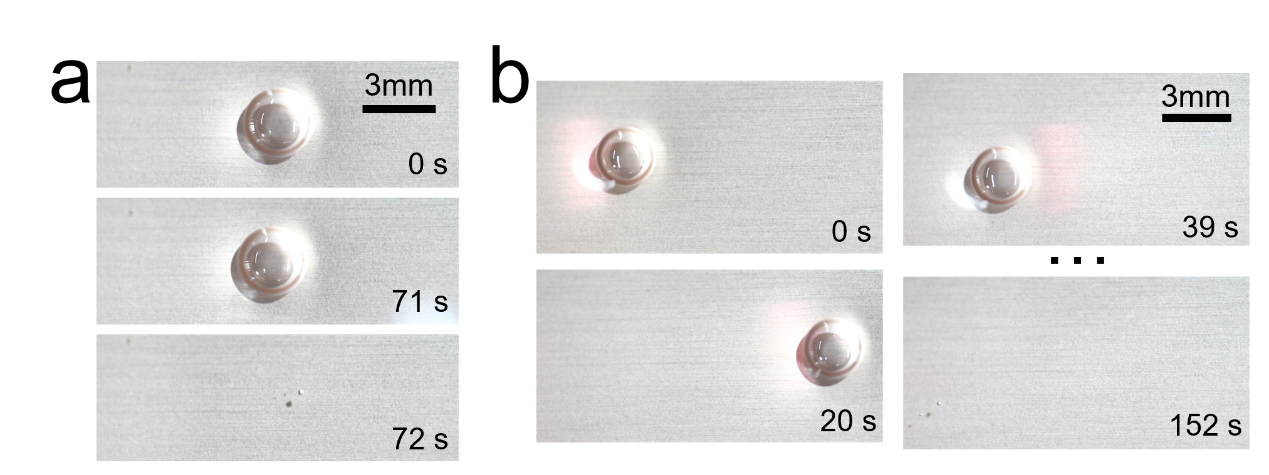


**Fig. S5.** **Comparison of bubble lifetime with and without laser-driven actuation.** (a) Time-sequence images showing the evolution of an ~8 µL gas bubble under corona discharge (4.6 kV) without laser irradiation. Under this condition, the bubble gradually shrinks and disappears within a maximum lifetime of approximately 72 s. (b) Time-sequence images showing the evolution of an ~8 µL gas bubble under the same applied voltage (4.6 kV) with laser-guided actuation. In this case, the bubble remains observable over repeated transport cycles, with a maximum lifetime of approximately 152 s. The extended bubble lifetime observed under laser-guided actuation may originate from the downward compression of the oil layer induced by the applied corona voltage, which increases the effective confinement of the bubble and delays its dissipation.


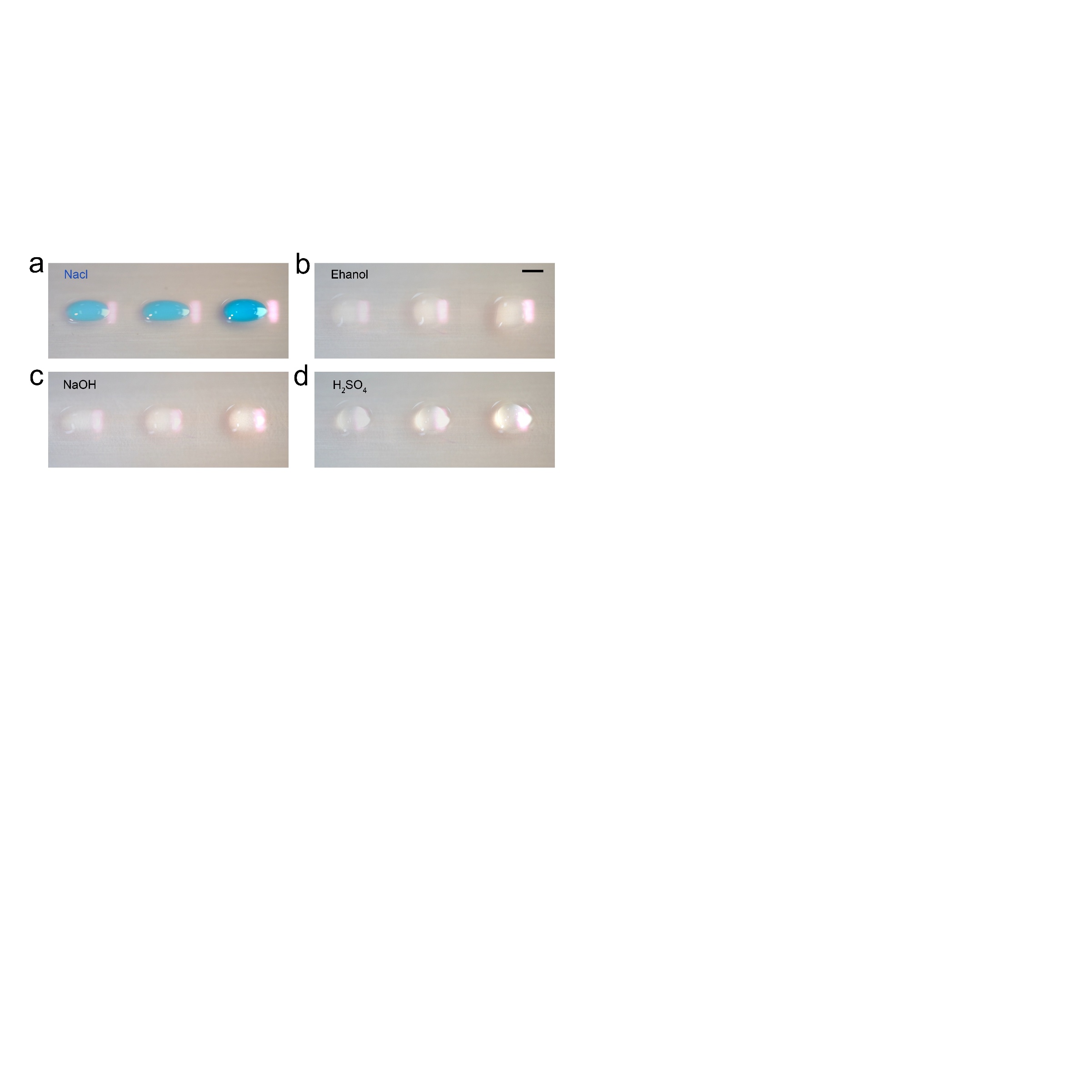


**Fig. S6. Photo-gated transport of multiple droplets.** Time-sequence images showing laser-guided transport of 10-µL droplets of (a) 5 M NaCl, (b) 75% ethanol, (c) 1 M NaOH, and (d) 1 M H₂SO₄ under an applied needle voltage of 6 kV.


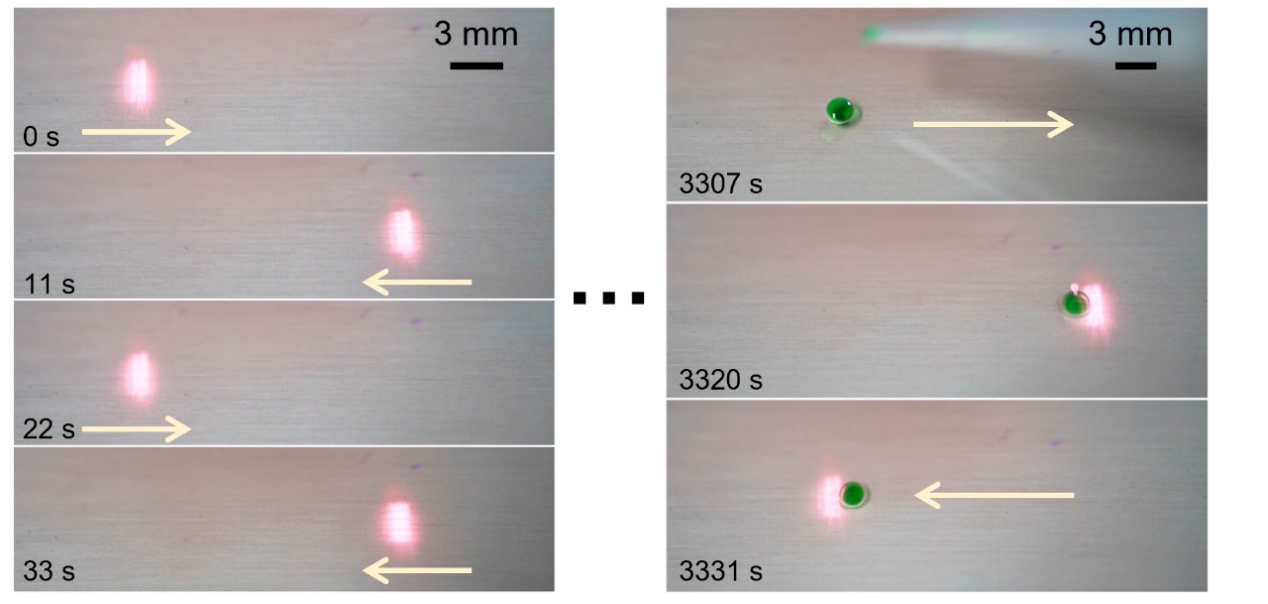


**Fig. S7.** Time-sequence images showing the actuation of an approximately 3 µL droplet on the PGCM platform after 150 laser back-and-forth scanning cycles at 6 kV. The droplet can still be guided to move along a predefined trajectory.


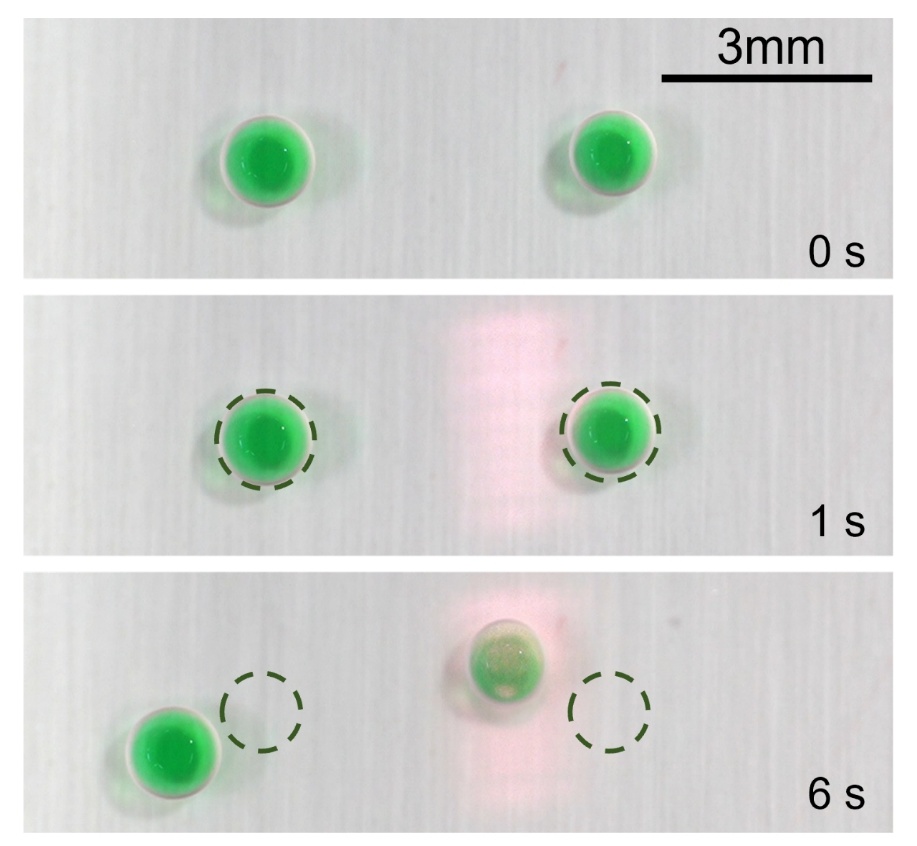


**Fig. S8. Attraction and repulsion behaviors inside and outside the binding radius** Two 2- µL droplets are placed at different initial positions relative to the photo-gated region under an applied needle voltage of 5 kV. When the droplet is located inside the binding radius (BR), it moves directionally toward the photo-gated region. In contrast, when the droplet is initially positioned outside the BR, it moves away from the laser spot.


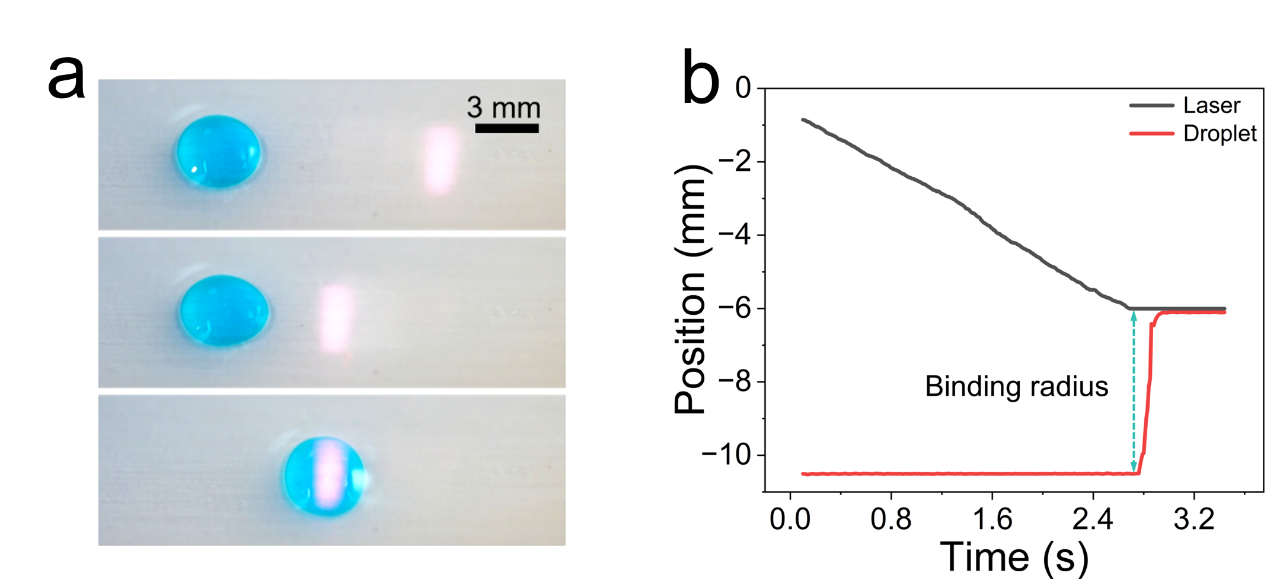


**Fig. S9. Experimental characterization of the binding radius** (a) Laser-approaching experiment performed on a 10 µL water droplet under an applied needle voltage of 7 kV. The needle electrode is positioned 4.5 cm above the substrate. (b) Temporal evolution of the positions of the droplet and the laser spot during the approaching process.

When the laser–droplet distance and the applied voltage are both relatively large, and the droplet remains nearly stationary or exhibits only very weak repulsive motion. As the laser spot gradually approaches and the distance decreases to a critical value, the droplet motion abruptly switches from weak repulsion (or near-stationary) to rapid directional attraction toward the laser spot. This critical distance is defined as the binding radius (BR).


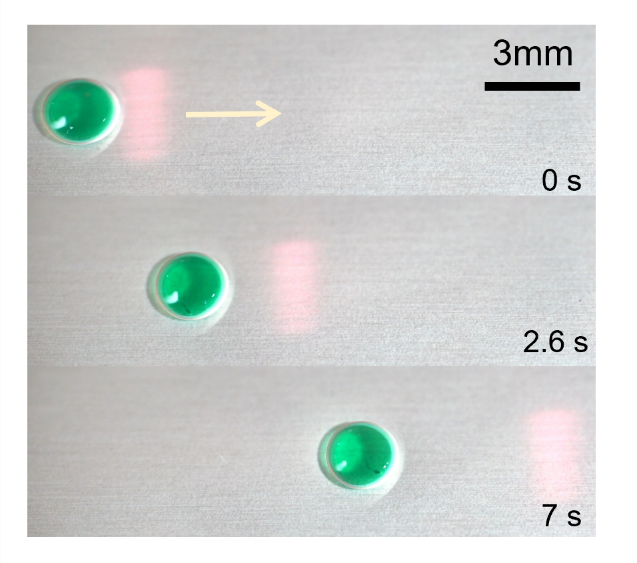


**Fig. S10. Measurement of the maximum stable droplet velocity using laser scanning.** The maximum stable droplet velocity is determined by gradually increasing the laser scanning speed. When the laser speed exceeds the droplet’s ability to follow, the droplet can no longer track the laser position, and the separation distance between the droplet and the laser spot increases with time. Representative time-sequence images acquired at an applied voltage of 6 kV are shown to illustrate the measurement procedure.


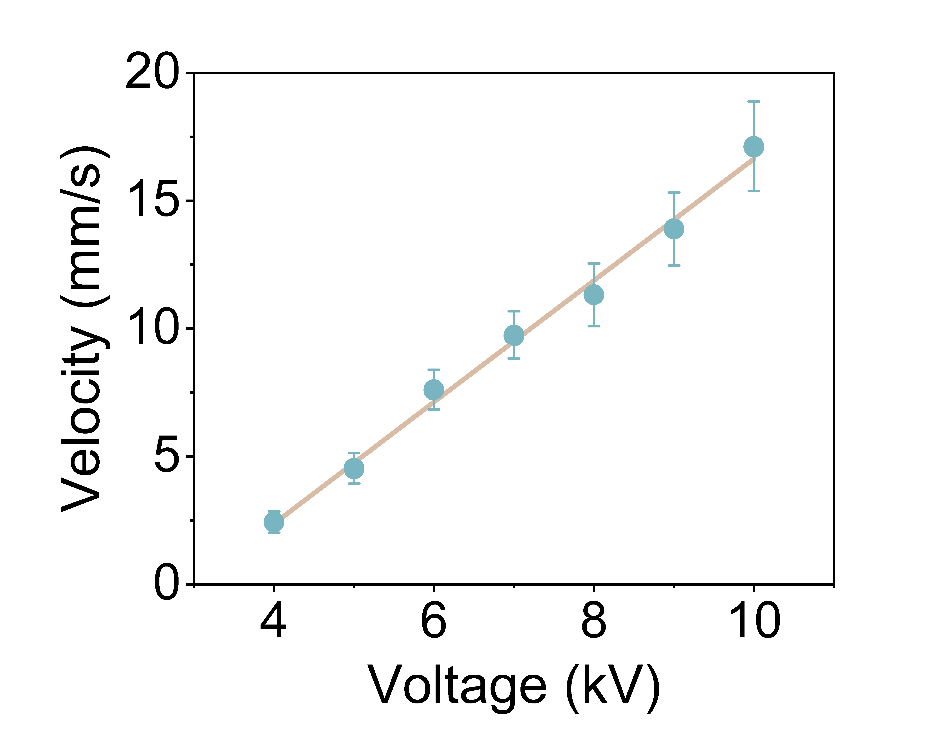
 u2i1

**Fig. S11. Dependence of the maximum stable droplet velocity on the applied voltage.** The maximum stable following velocity of a 10 µL droplet is measured under different applied needle voltages, with the needle positioned 4.5 cm above the substrate. The results show that the maximum droplet velocity increases with increasing applied voltage, reaching approximately 17.6 mm s⁻¹ at 10 kV.


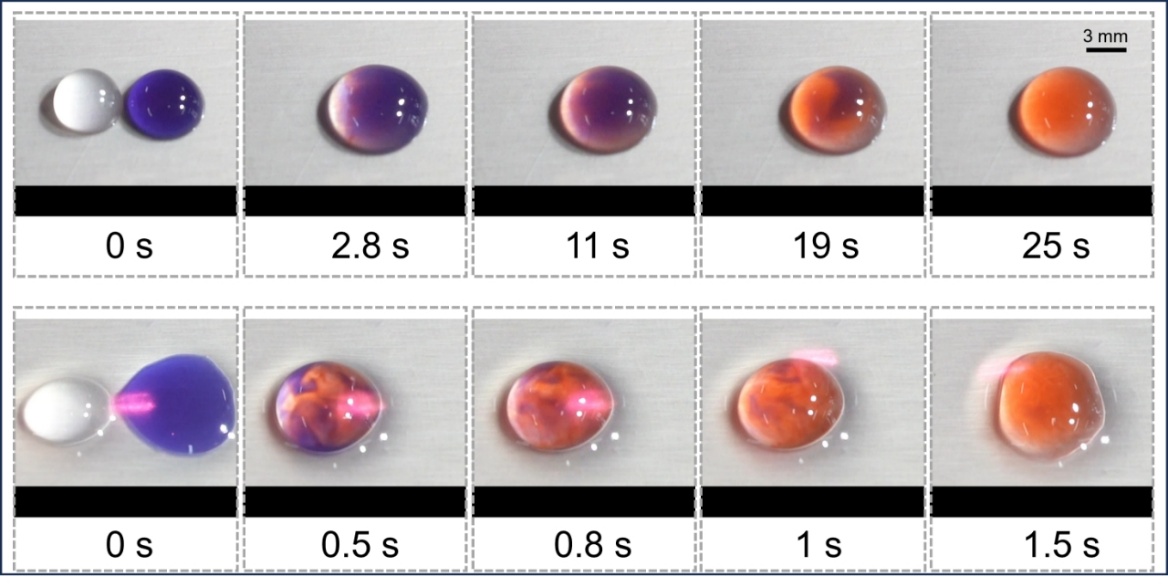


**Fig. S12. Comparison of droplet mixing and reaction with and without photo-gated actuation. Time-sequence images comparing the reaction process between a 10 µL hydrochloric acid solution (1 M) and a 10 µL litmus solution (10 g L⁻¹) under different conditions. Top row: reaction process without laser irradiation and without applied voltage, where the two droplets merge slowly and the color transition completes after approximately 25- s. Bottom row: reaction process with simultaneous laser irradiation and applied voltage, where the droplets rapidly mix and the color transition completes within approximately 1.5 s.**


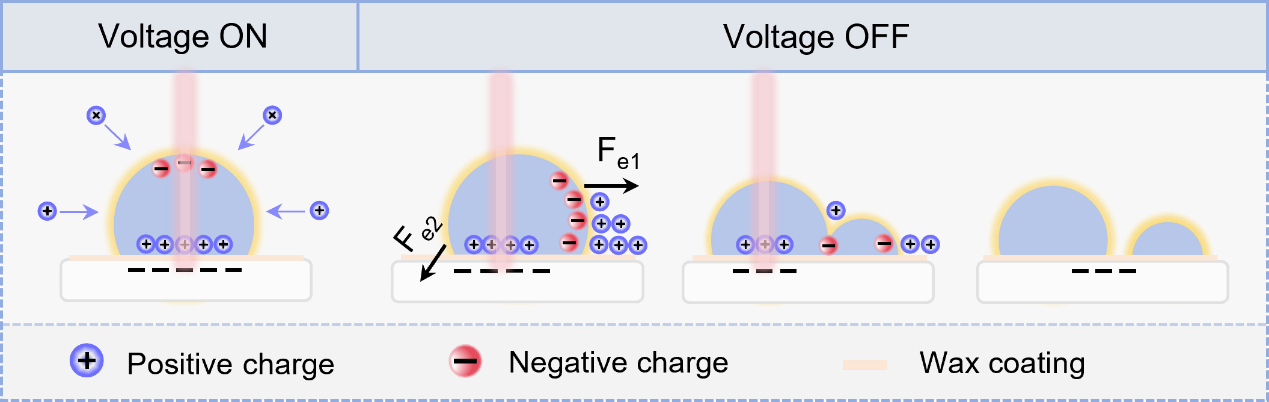


**Fig. S13. Mechanism of droplet splitting induced by a photo-gated electric field.**The splitting process is driven by **charge separation and electrostatic stretching within the droplet, initiated by a laterally inhomogeneous electric field**. This field is dynamically created by the specific combination of terminating the corona discharge while keeping the laser illumination active. The key steps are as follows:

1. **Formation of a Lateral Electric Field Gradient**: When the corona discharge is off, the pre-charged droplet retains a net charge. Simultaneous laser illumination locally melts the wax layer, creating a grounded conductive region directly beneath the droplet. This establishes a sharp lateral gradient in surface potential between this grounded zone and the surrounding insulated, charged area, generating a strong in-plane electric field.
2. **Charge Separation and Droplet Polarization**: The conductive droplet responds to this lateral field. Free ions inside the droplet redistribute, with counter-ions accumulating on opposite sides. This leads to an induced dipole or higher-order polarization aligned with the field.
3. **Electrostatic Stretching**: The grounded region attracts charges of opposite polarity within the droplet, while the charged non-illuminated area attracts charges of the same polarity (via image charge effects). These opposing electrostatic attractions act on different sides of the droplet, applying a tensile stress that elongates it along the field direction.
4. **Instability and Pinch-Off**: As the droplet is stretched, a thin liquid neck forms. The electrostatic pressure concentrated at the neck competes against the restoring force of surface tension. Splitting occurs when the electrostatic stretching force overcomes the surface tension, leading to the instability and rupture of the neck.


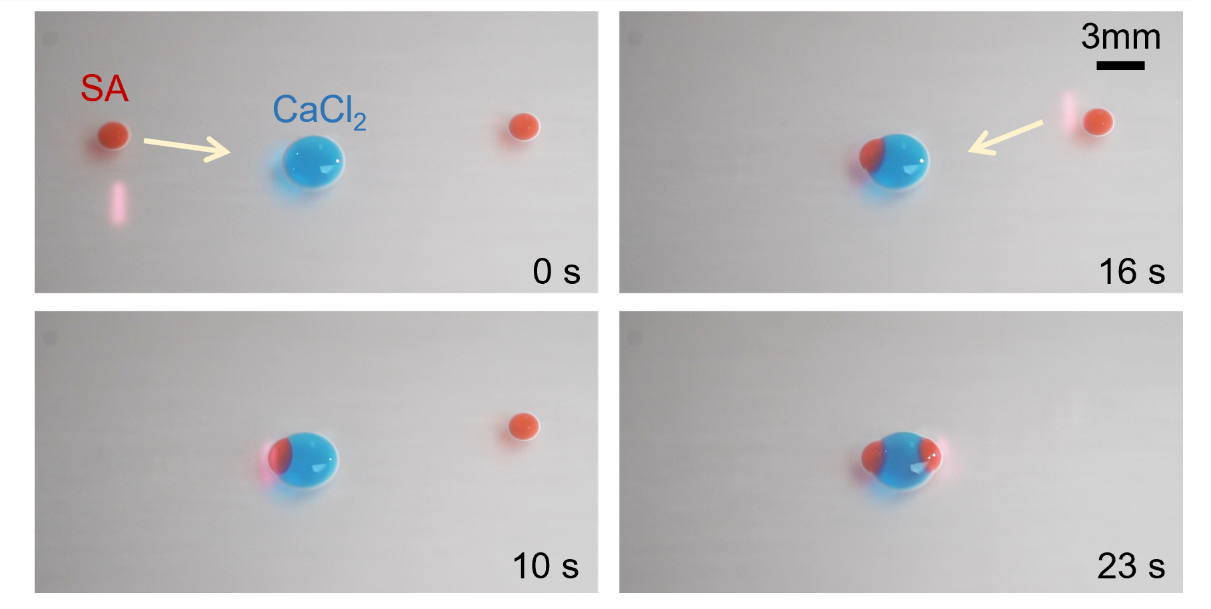


**Fig. S14. Programmable assembly of multiple droplets into a molecule-like configuration.**

**Three droplets are guided under photo-gated control to form a spatial configuration resembling a water molecule. A central CaCl₂ droplet (10 µL) is positioned between two sodium alginate (SA) droplets (3 µL each) under an applied needle voltage of 4 kV. The experiment is conducted in 50 cSt silicone oil, with the droplets fully immersed in the oil layer (thickness ≈ 5 mm).**


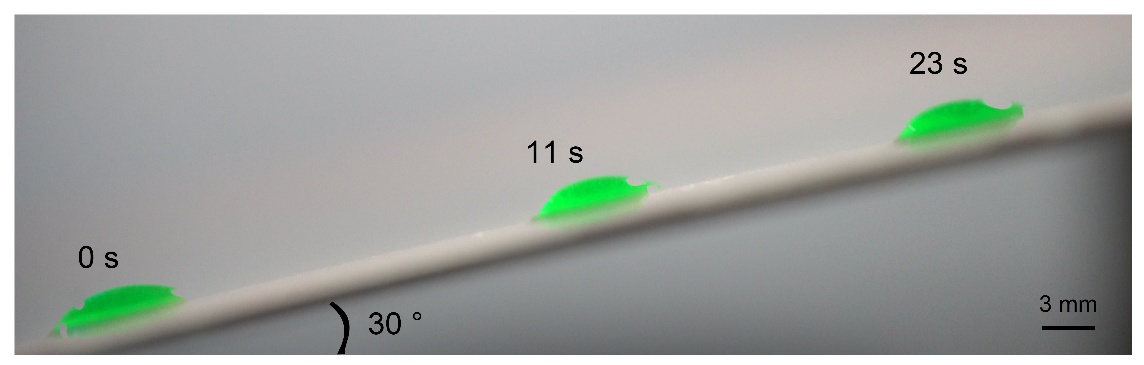


**Fig. S15.** **Transport of a 10 µL droplet on a planar surface tilted at an angle of 30°. The droplet is guided along the surface under photo-gated actuation, demonstrating directional motion on an inclined substrate with zero Gaussian curvature.**


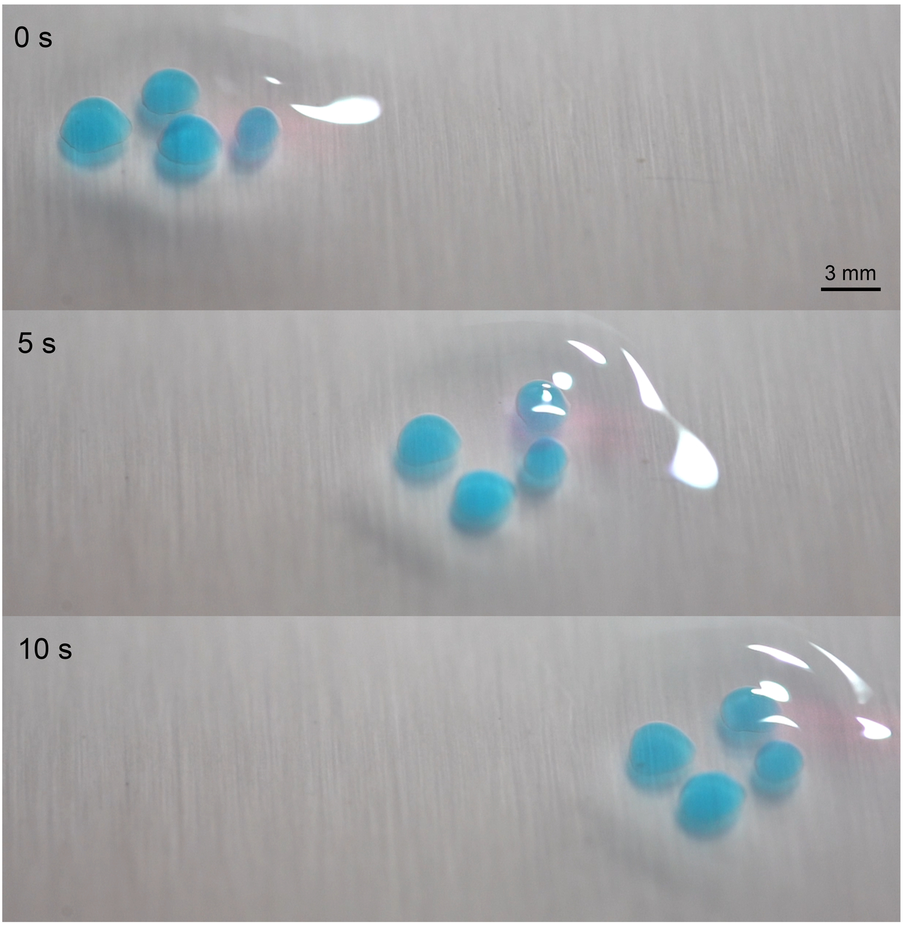


**Fig. S16. Synchronous transport of 4 droplets under a single photo-gated field. Multiple droplets (two 1.8 µL droplets, one 1.4 µL droplet, and one 1.0 µL droplet) are simultaneously guided under an applied needle voltage of 5 kV. All droplets move toward the photo-gated region and migrate synchronously with the laser spot. The experiment is conducted in 50 cSt silicone oil, with the droplets fully immersed in an oil layer of approximately 2 mm thickness.**


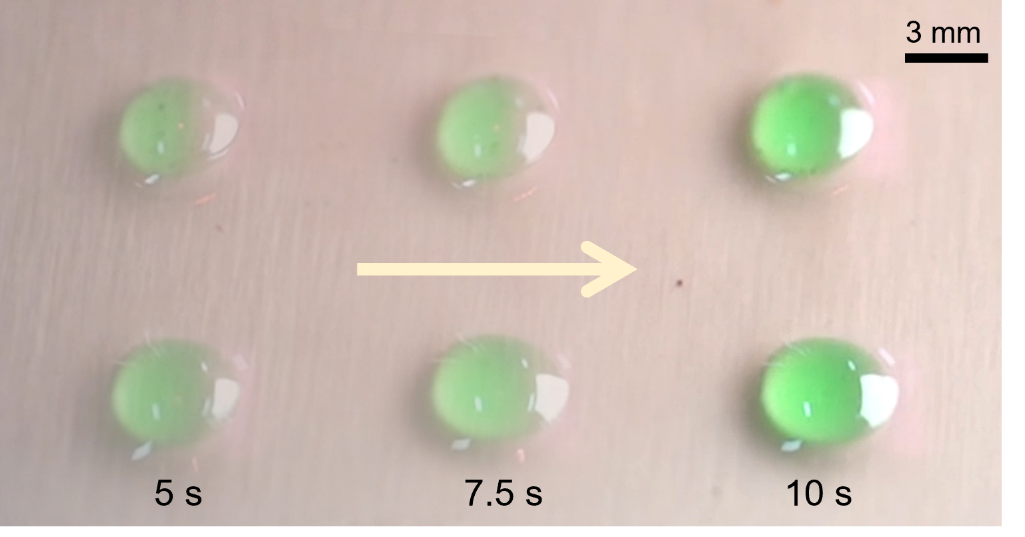


**Fig. S17. Parallel manipulation of two droplets using dual laser spots.** Two independent laser spots are employed to simultaneously actuate two droplets with identical volumes of 5 µL. Each droplet is guided directionally by its corresponding laser spot, and no noticeable interference between the two droplets is observed during parallel transport.


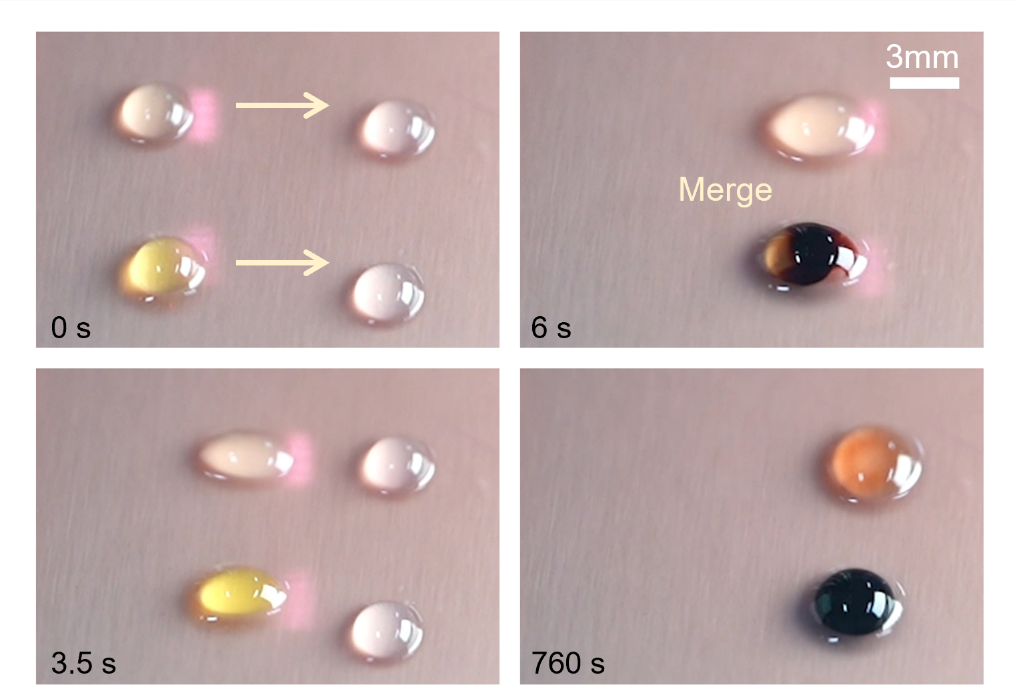


**Fig. S18. Parallel chemical reactions and detection under dual-laser actuation.** Two closely spaced laser spots are used to independently drive two sets of 5 µL droplets on the same PGCM platform. One reaction involves a mixed droplet of starch solution (0.5 wt%) and hydrogen peroxide (2.94 mol·L⁻¹) merging with a potassium iodide solution (1 mol·L⁻¹). The other reaction involves a ferric chloride solution (0.1 mol·L⁻¹) merging with a KSCN solution (0.05 mol·L⁻¹). Under the guidance of their respective laser spots, both droplet pairs undergo directed transport, merging, and reaction, accompanied by observable color changes.


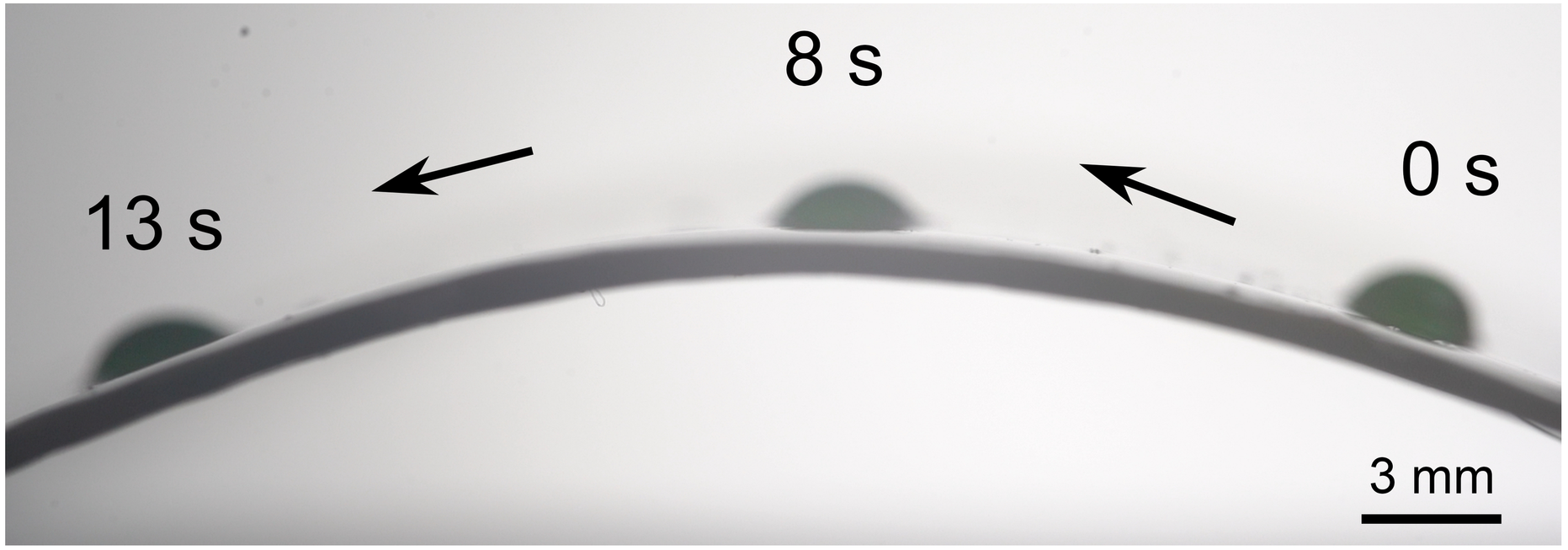


**Fig. S19. Droplet actuation on a deformable surface with zero Gaussian curvature. A deformable substrate with zero Gaussian curvature is constructed by mechanically bending the base plate. A 2.5 µL droplet is actuated on the curved surface under photo-gated control. The droplet remains directionally movable after substrate deformation and follows the laser-defined trajectory along the curved surface.**


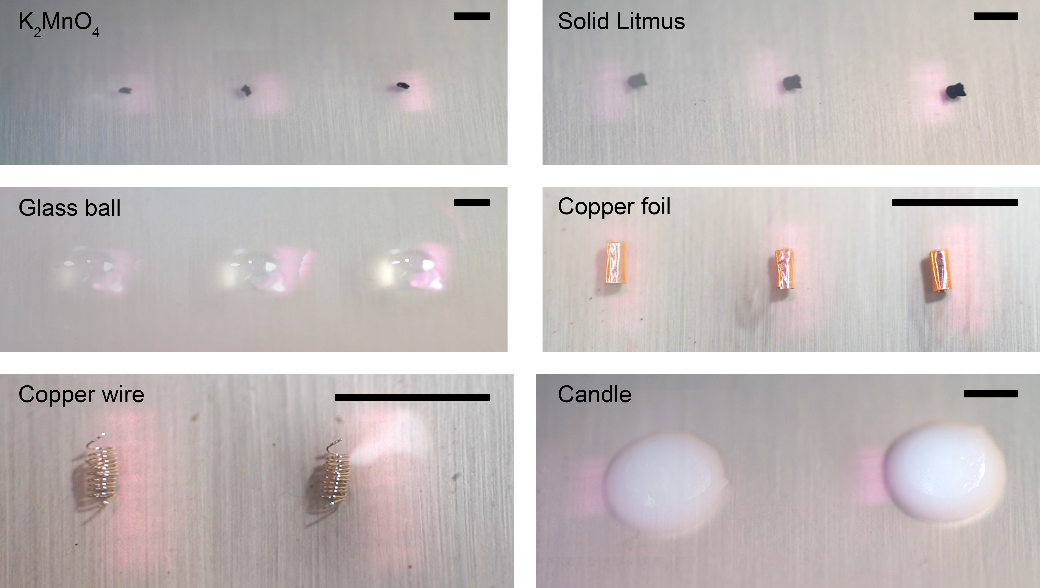


**Fig. S20. Unified photo-gated transport of solid objects with different shapes and properties. Under an applied needle voltage of 6 kV, the PGCM platform drives a variety of solid objects with different shapes, sizes, and material properties. The transported objects include irregular potassium permanganate crystals, solid litmus particles, spherical glass beads (~2.5 mm in diameter), cylindrical copper foils (~0.8 mm in base diameter and ~1.4 mm in length), wire-shaped copper segments (~0.1 mm in diameter and ~1 mm in length), and disk-shaped candle wax solids (~4 mm in diameter). All solid objects exhibit directional motion toward the photo-gated region.** Scale bar: 3 mm


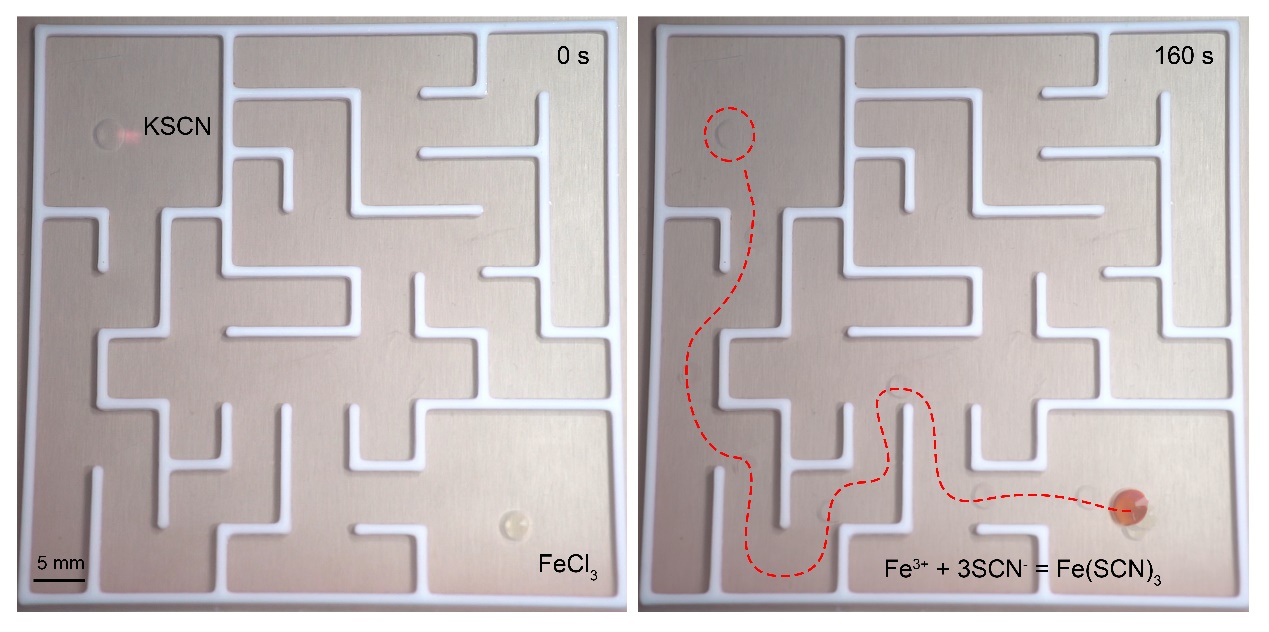


**Fig. S21. Programmable droplet navigation and in situ chemical reaction in a maze structure. At 5 kV, A 10 µL KSCN droplet (0.05 mol**·**L⁻¹) follows the laser-defined path through the maze under electric-field confinement and subsequently merges with a 10 µL FeCl₃ droplet (0.1 mol**·**L⁻¹), triggering a colorimetric reaction (Fe³⁺ + 3SCN⁻ → Fe(SCN)₃). The experiment is conducted in 50 cSt silicone oil, with the droplets fully immersed in an oil layer of approximately 4 mm thickness.**


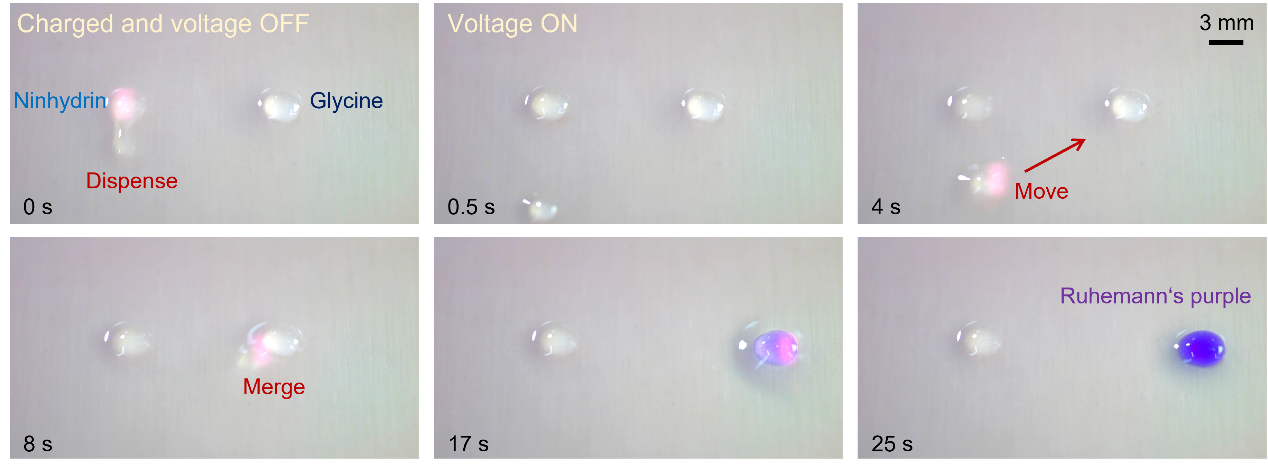


**Fig. S22. Sequential droplet splitting, transport, and colorimetric reaction triggering. At 5 kV, a 5 µL droplet of 1 wt% ninhydrin solution is first charged and then partially split after the applied voltage is turned off. The voltage is subsequently reapplied to drive the separated small droplet toward a 5 µL glycine solution (5 wt%). After merging, a colorimetric reaction is triggered, and the merged droplet gradually turns purple.**


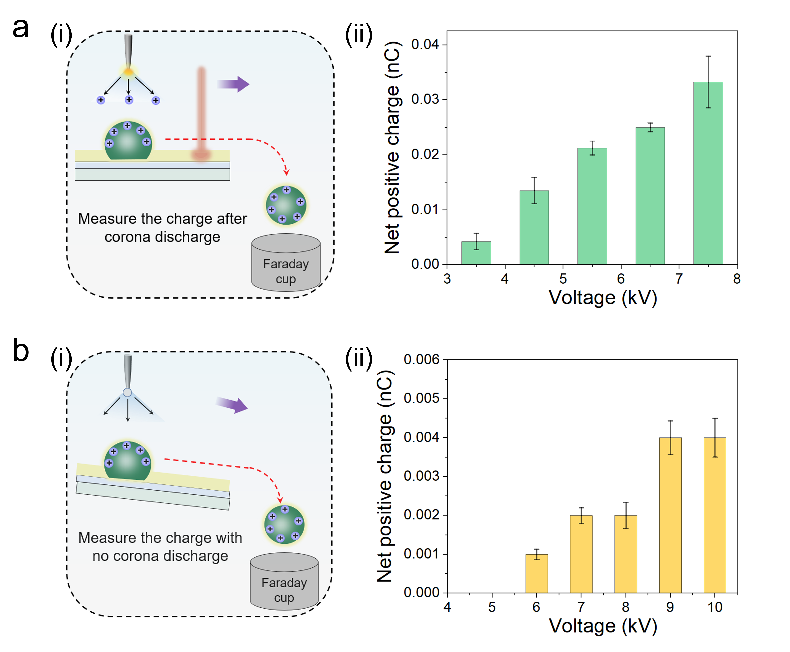


**Fig. S23. Charge characterization of droplets under corona discharge versus pure electrostatic polarization.** (a) Measurement of net charge injected by corona discharge. (i) Schematic of the experimental setup: a tungsten needle electrode was positioned 4.5 cm above the substrate, and a positive DC voltage (3.5–7.5 kV) was applied to sustain stable corona discharge for 1 min. A 10 µL deionized water droplet was subsequently transported into a Faraday cup connected to a nanocoulomb meter, enabling quantification of the net charge originating from air-ionization-driven charge injection. (ii) Voltage-dependent accumulation of injected net charge. (b) Measurement of residual charge under pure polarization. (i) Schematic of the experimental setup: the needle electrode was wrapped with a polyimide insulating layer to suppress corona discharge while preserving the same non-uniform electric field geometry. A 10 µL deionized water droplet was exposed to this field for 1 min to allow full polarization. The voltage was then turned off, and the substrate was gently tilted to transfer the droplet into the Faraday cup by gravity, ensuring the absence of contact charging and residual field effects during measurement. (ii) Voltage-dependent residual charge under polarization-only conditions.


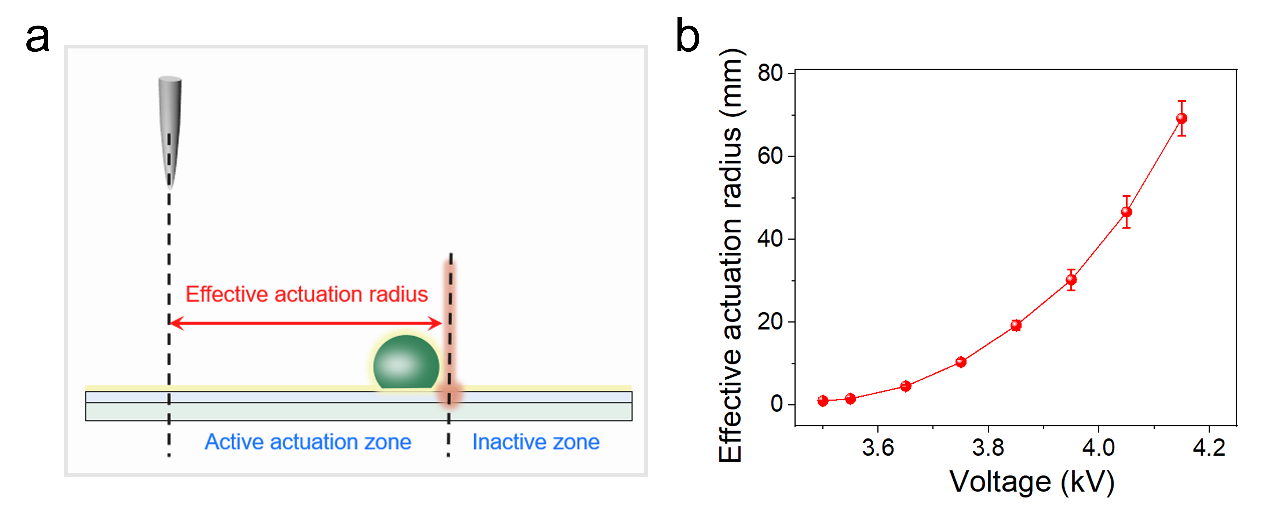


**Fig. S24.** Characterization of the lateral effective zone in the PGCM platform. (a) Schematic illustration showing the effective actuation radius, defined as the maximum horizontal distance from the needle axis at which a droplet can be reliably driven. (b) Measured effective actuation radius as a function of applied voltage at a needle-to-substrate separation of 4.5 cm.


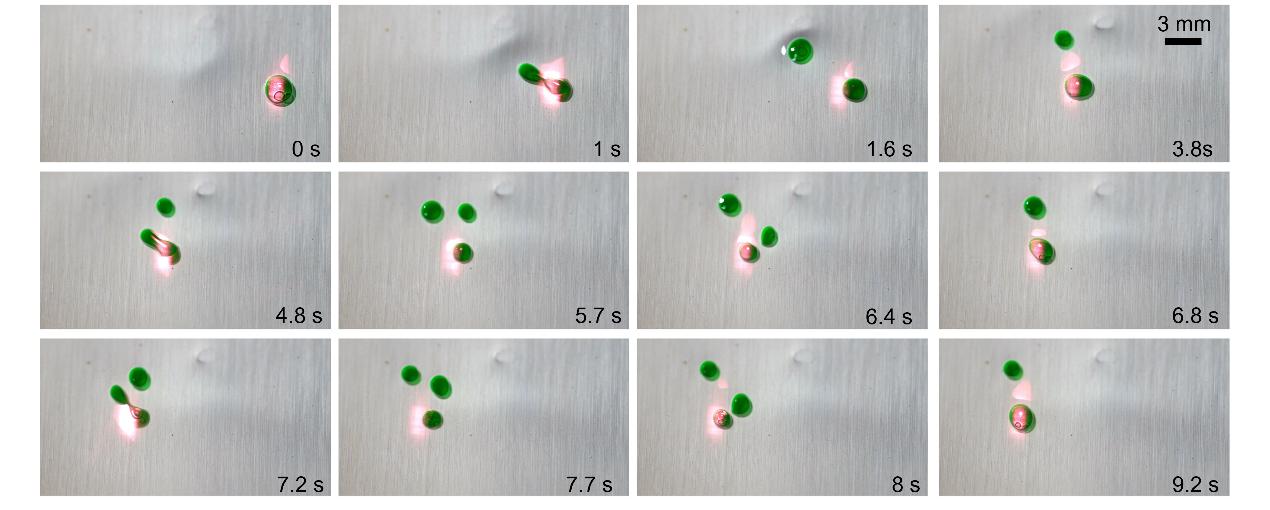


**Fig. S25.** Droplet dynamics under inverted electric field polarity. Time-sequence images showing the cyclic fission and coalescence of a 10 µL droplet driven at 8 kV with the stainless-steel substrate connected to the positive high-voltage terminal and the needle electrode grounded.。


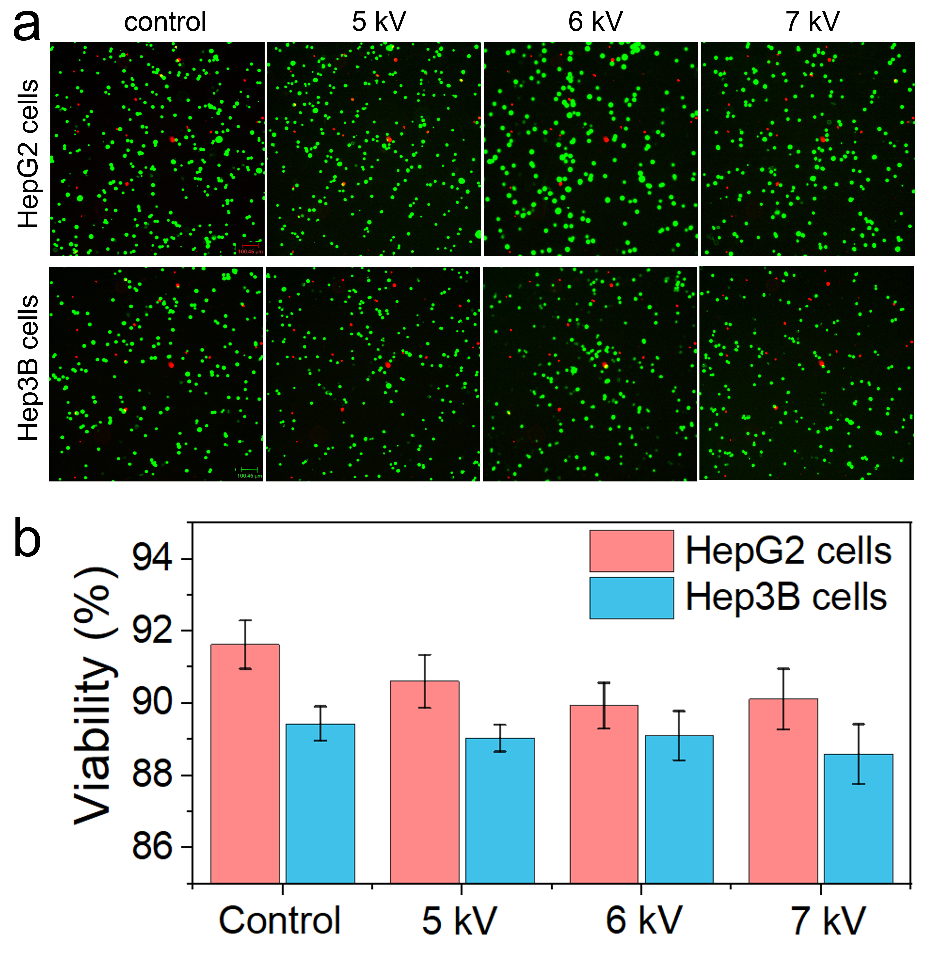


**Fig. S26**. Live/dead fluorescence assay of HepG2 and Hep3B cells after PGCM manipulation. (a) Fluorescence micrographs showing Calcein-AM (green, live) and PI (red, dead) staining under control and PGCM-actuated conditions (5–7 kV). (b) Cell viability quantification across all tested voltages. Error bars represent standard deviations from three replicates.


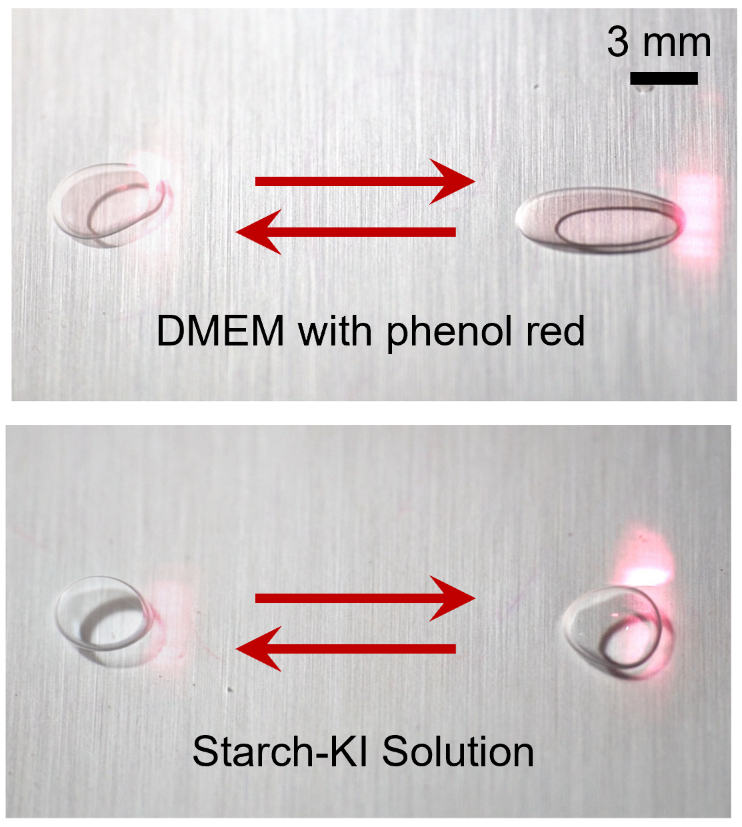


**Fig. S27**. Control experiments assessing chemical stability of biological droplets under PGCM manipulation. (a) pH stability test. A 5 µL droplet of phenol red–supplemented cell culture medium (phenol red: 0.015 g L⁻¹; initial pH ~7.4) was manipulated on the PGCM platform at 7 kV for 5 min. Phenol red undergoes a distinct color transition from red (pH ~7.4) to yellow (pH < 6.8) upon acidification. No visible color change was observed after manipulation, indicating that the oil encapsulation effectively prevents acidification by dissolved ionic species or electrochemical byproducts. (b) Reactive species (ozone) penetration test. A 5 µL droplet of starch–potassium iodide solution (1 wt% starch, 0.1 M KI) was manipulated at 7 kV for 2 min. In the presence of ozone or other oxidizing species, iodide is oxidized to iodine, which forms a characteristic blue-black complex with starch. The droplet remained completely clear after manipulation, confirming that reactive species generated in the air gap do not penetrate the oil encapsulation to reach biologically significant concentrations within the aqueous droplet.


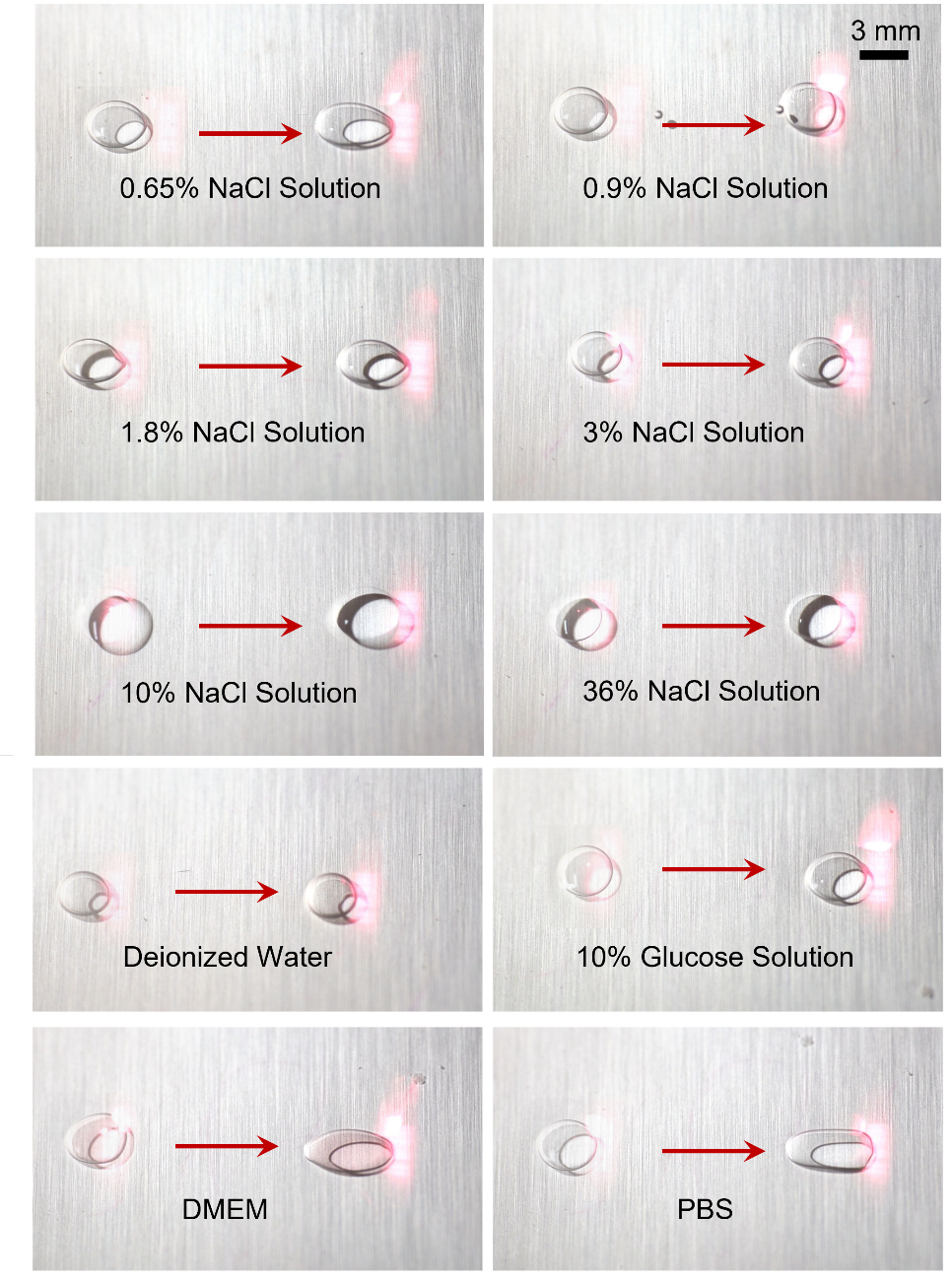


**Fig. S28**. Transport of 10 µL droplets of biologically relevant solutions on the PGCM platform. (a) Deionized water, 10% glucose, DMEM, PBS, and graded NaCl solutions (0.65%–36% w/v) were all successfully actuated. Red arrows indicate the direction of motion. Scale bar: 3 mm.


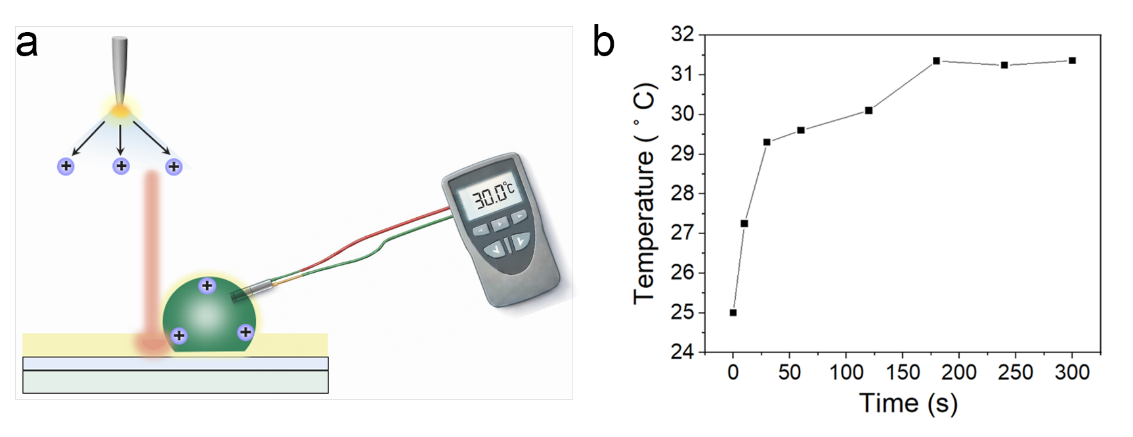


**Fig. S29.** Local temperature measurement during laser actuation. (a) Schematic of the experimental setup showing a K-type thermocouple inserted into a 10 µL droplet held stationary at the laser spot under 7 kV. (b) Temporal evolution of droplet temperature under continuous 808 nm laser irradiation (1 W, 300 mW mm⁻²). The temperature rises from ~25 °C to a plateau of ~31 °C within ~3 min, stabilized by convective cooling from the silicone oil layer and conductive heat dissipation to the temperature-controlled stage.

**Table S1**. Density and volume range of objects manipulated by the PGCM

| Phase | Material | Density (g/cm³) | Operable volume/size range | Notes |
| --- | --- | --- | --- | --- |
| Gas | Air bubble | ~0.001 | 40 nL – 42 μL | Stable manipulation in oil phase |
| Liquid | Deionized water | 1.00 | 1 pL – 10 mL | Benchmark system |
|  | 75% ethanol | 0.87 | 0.1 – 500 μL | Low-surface-tension liquid |
|  | H₂SO₄  (1 mol·L⁻¹) | 1.06 | 0.5 – 1000 μL | Strong electrolyte |
|  | NaOH  (1 mol·L⁻¹) | 1.04 | 0.5 – 1000 μL | Strong electrolyte |
|  | H_2_O_2_  (3.6 mol·L⁻¹) | 1.03 | 0.5 – 1000 μL | Oxidant |
|  | Mineral oil | 0.84 | 0.1 – 500 μL | Nonpolar liquid |
|  | Bromothymol blue solution  (0.04 wt %) | 1.001 | 0.5 – 1000 μL | Organic indicator dye |
|  | Litmus solution (0.1wt%) | 1.001 | 0.1 – 100 μL | Indicator solution |
| Solid | Glass ball | 2.49 | 0.5 – 2.5 mm diameter | Inorganic (glass) |
|  | POM particle | 1.42 | 0.5 – 7 mm diameter | Polymer |
|  | Candle particle | 0.91 | 0.5 – 5 mm diameter | Organic wax |
|  | KMnO₄ | 2.70 | 50 – 400 μm diameter | Inorganic salt crystal |
|  | Solid litmus | N/A (mixture) | 50 – 800 μm diameter | Natural dye mixture |
|  | Cu particle | 8.96 | 20 – 100 μm diameter | Metal |
|  | Carbon particle | ~1.8 | 20 – 500 μm diameter | Irregular shapes tested |

**Table S2.** Scoring Criteria Definition

| **Dimension** | **Y (Yes)** | **M (Medium)** | **N (No)** |
| --- | --- | --- | --- |
| **Environment adaptability** | Demonstrated actuation on **≥2 non-planar orientations** (e.g., vertical, inverted, or curved) | Demonstrated on **1 non-planar orientation** (e.g., inclined or vertical only) | **Planar only**; no demonstrated capability on tilted, vertical, inverted, or curved surfaces |
| **Functional diversity** | **≥3 core operations** (transport, merge, split/dispense) demonstrated | **2 core operations** demonstrated | **≤1 core operation** or restricted to simple transport only |
| **System simplicity** | **No lithographic patterning, microfabricated electrodes, or complex microstructure substrate coatings** (e.g., ferroelectric wafers, pyroelectric crystals, superhydrophobic layers, or superlubricating layers). | **No patterned electrodes**, but requires specialized functional layers (e.g., pyroelectric films, ferroelectric polymers) | **Requires complex microfabrication** (e.g., electrode arrays, ferroelectric substrates, or material-specific functionalization) |
| **Multiphase versatility** | Demonstrated manipulation of **liquids, solids, and gas bubbles** without surface modification | Demonstrated manipulation of **2 phases** (e.g., liquid + solid, or liquid + bubble) | **Single phase only** (e.g., only liquids or only solids) |

**Table S3.** Material costs

| **Material** | **Specification** | **Cost** |
| --- | --- | --- |
| Laser | 1 W | 607**￥** |
| Stainless steel Plate | 100mm×100mm×0.5mm | 7**￥** |
| Paraffin | Φ12mm×160mm | 0.6**￥** |
| Silicone oil | 50cSt | 28**￥**/kg |
| Dye | **——** | 3**￥**/100g |
| Acrylic box | 110mm×110mm×30mm | 4**￥** |
| Copper wire | Φ3mm×1000mm | 3**￥** |
| Temperature control platform | 12V20A | 258**￥** |
| Insulating tape | 10000mm×16mm×0.2mm | 1.2**￥** |

**Supporting Movies:**

Movie S1. Multimodal programmable transport on the PGCM platform.

Movie S2. Comparison of bubble lifetime with and without laser-driven actuation.

Movie S3. Durability of the phase-change layer over 150 melt–freeze cycles with stable actuation.

Movie S4. Droplet shuttling and tracking under laser scanning

Movie S5. Tracking failure beyond the maximum laser scanning speed

Movie S6. Directed droplet navigation, fusion reaction, and corona-off triggered splitting.

Movie S7. Symmetric droplet splitting induced by central laser illumination.

Movie S8. Recursive multi-step splitting of a residually charged droplet under continuous illumination.

Movie S9. Anti-gravity droplet transport in an inverted configuration.

Movie S10. Capturing a freely falling droplet on a vertical plate and reversing its motion.

Movie S11. Path-guided droplet transport on a positively curved hemispherical surface.

Movie S12. Cross-scale droplet manipulation spanning 1 pL to 10 mL.

Movie S13. Manipulation of solid particles and bubbles across sizes

Movie S14. Shuttle-transport-enabled enrichment from oil to water

Movie S15. Bubble capture and fusion of 5 µL and 8.5 µL bubbles

Movie S16. Solid–liquid–gas tri-phase sorting into Region 1, Region 2, and Region 3.

Movie S17. Laser-enhanced solid–liquid reaction between a Cu foil and an HNO3 droplet with accelerated gas generation.

Movie S18. Residue-free “AUST” pattern writing using a 2 µL fluorescent droplet.
